# A hierarchical process model links behavioral aging and lifespan in *C*. *elegans*

**DOI:** 10.1371/journal.pcbi.1010415

**Published:** 2022-09-30

**Authors:** Natasha Oswal, Olivier M. F. Martin, Sofia Stroustrup, Monika Anna Matusiak Bruckner, Nicholas Stroustrup

**Affiliations:** 1 Centre for Genomic Regulation (CRG), The Barcelona Institute of Science and Technology, Barcelona, Spain; 2 Universitat Pompeu Fabra (UPF), Barcelona, Spain; UNITED STATES

## Abstract

Aging involves a transition from youthful vigor to geriatric infirmity and death. Individuals who remain vigorous longer tend to live longer, and within isogenic populations of *C*. *elegan*s the timing of age-associated vigorous movement cessation (VMC) is highly correlated with lifespan. Yet, many mutations and interventions in aging alter the proportion of lifespan spent moving vigorously, appearing to “uncouple” youthful vigor from lifespan. To clarify the relationship between vigorous movement cessation, death, and the physical declines that determine their timing, we developed a new version of the imaging platform called “The Lifespan Machine”. This technology allows us to compare behavioral aging and lifespan at an unprecedented scale. We find that behavioral aging involves a time-dependent increase in the risk of VMC, reminiscent of the risk of death. Furthermore, we find that VMC times are inversely correlated with remaining lifespan across a wide range of genotypes and environmental conditions. Measuring and modelling a variety of lifespan-altering interventions including a new RNA-polymerase II auxin-inducible degron system, we find that vigorous movement and lifespan are best described as emerging from the interplay between at least two distinct physical declines whose rates co-vary between individuals. In this way, we highlight a crucial limitation of predictors of lifespan like VMC—in organisms experiencing multiple, distinct, age-associated physical declines, correlations between mid-life biomarkers and late-life outcomes can arise from the contextual influence of confounding factors rather than a reporting by the biomarker of a robustly predictive biological age.

## Author summary

Aging produces a variety of outcomes—declines in various measures of health and eventually death. By studying the relationship between two outcomes of aging in the same individual, we can learn about the underlying aging processes that cause them. Here, we consider the relationship between death and an outcome often used to quantify health in *C*. *elegans*—vigorous movement cessation which describes the age-associated loss of an individuals’ ability to move long distances. We develop an automated imaging platform that allows us to precisely compare this pair of outcomes in each individual across large populations. We find that individuals who remain vigorous longer subsequently have a shorter remaining lifespan—a pattern that holds even after vigorous movement and lifespan timing are both altered by several different mutations and interventions in aging. Modelling our data using a combination of simulation and analytic studies, we demonstrate how the relative timing of vigorous movement cessation and death suggest that these two outcomes are driven by distinct aging processes. Our data and analyses demonstrate how two outcomes of aging can be correlated across individuals with the timing of one predicting the timing of the other, but nevertheless be driven by mostly distinct underlying physical declines.

## Introduction

Aging produces a wide distribution of lifespan, and only a small amount of variation in lifespan is determined by heritable factors [1]. Searching for non-genetic factors influencing lifespan, researchers have identified a diverse array of biomarkers that predict death times [2–5], many of which are informative when measured early in adulthood. Little is known, mechanistically, about the processes that link inter-individual physiologic variation early in life to the timing of outcomes happening decades later. However, a general dynamics of aging seems to be shared across taxa, in which individuals start adulthood relatively synchronous in respect to many biomarkers but then diverge over time as some individuals age faster than others [2, 6–10]. The dynamics of aging can measured using a wide range of biomarkers, including for example gene regulation [11], chromatin modification [12], and serum markers [13], all of which can serve as useful proxies for the underlying dynamics of aging.

Yet, the major challenge in aging biomarker research remains that, though many biomarkers correlate with each other and with outcomes, these correlation are always less than perfect [14, 15]. Discordant predictions by different biomarkers raises conceptual questions: how should we interpret situations where an individual is predicted by one biomarker to be young and by another to be old? Such ambiguity is heightened by the discovery that many mutations and interventions in aging alter the quantitative relationships between biomarkers and the outcomes they predict. Several mutations and interventions in aging have been shown to produce broad, concordantly beneficial effects across multiple age-associated phenotypic changes, but others have been found to produced mixed effects, for example increasing lifespan disproportionately to changes in age-associated declines in behavior [4, 6, 16, 17]. To explain the discordant predictions made by different biomarkers and their complex, context-dependent relationships with functional outcomes in aging, more rigorous models are needed to describe the causal relationships between biomarkers the underlying aging processes on which they report.

Here, we focus on an important behavioral biomarker of *C*. *elegans* lifespan: vigorous movement cessation (VMC). *C*. *elegans* start adulthood vigorously exploring their environment but over time gradually slow and eventually stop crawling. Most *C*. *elegans* housed in standard laboratory conditions remain alive but non-motile for approximately the final 10% of their lives [18]. In this sense, vigorous movement cessation in nematodes is analogous to the loss of mobility seen in older humans, in which the elderly often spend the last several years of their life unable to walk unassisted. Both in humans and *C*. *elegans*, late-life reductions in motility represent unambiguous, macroscopically observable outcomes of complex, progressive failures of neuromuscular function [7, 19–21]. A potent biomarker of lifespan, the timing of an individual’s VMC strongly correlates with their death time [4, 7, 18, 22]. In nematodes, VMC is often used as a proxy for organismal “health”, defining a “healthspan” that describes a period of youthful vigor that is followed by a period of “decrepitude” ending in death.

Despite VMC’s power to predict lifespan within isogenic populations, several studies have demonstrated that the relationship between VMC times and lifespan is quite malleable. Many mutations and interventions in aging alter VMC disproportionately to lifespan, and vice versa, creating animals that live longer but suffer an extended “decrepit” period at the end of life [4, 17, 23–27]. These findings call into question the predictive power of VMC as a biomarker, as the relationship between VMC and lifespan observed within each population does not appear to remain constant across genotypes and after interventions. To better understand the relationship between VMC, death, and the age-associated physical declines that determine their timing, we set out to characterize their relationship using combination of improved measurement techniques and formal quantitative modeling.

## Results

### The lifespan machine can collect behavioral and lifespan data in very large populations

We reasoned that the automated microscopy platform dubbed “the lifespan machine” [28] could be developed to jointly measure VMC times and lifespan at unprecedented statistical resolution and scale. The “lifespan machine” is an imaging system that combines an array of modified flatbed document scanners to image several square meters of nematode at eight-micron resolution. Using this platform, tens of thousands of nematodes can be individually and simultaneously imaged in time courses lasting a month or more. The lifespan machine collects data at lower temporal resolution than other ageing-focused imaging approaches [6, 7, 24], only once every hour, but it produces data at a higher precision across much larger populations. Therefore, the technology is well suited a systematic comparison of VMC and lifespan.

**Fig 1:**
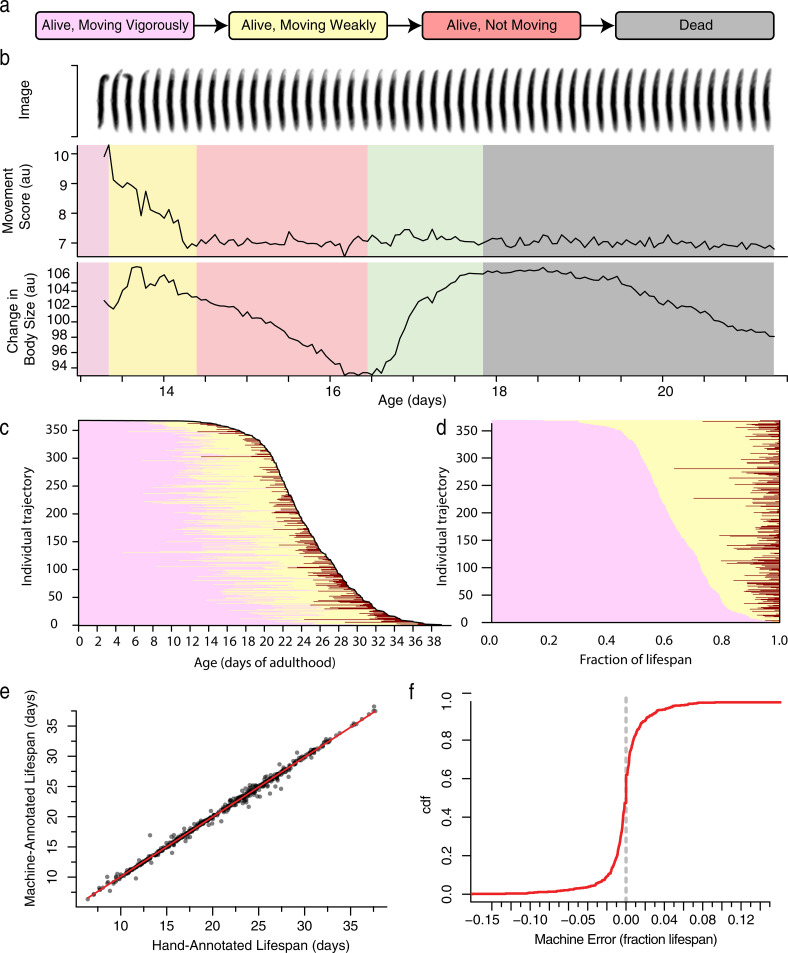
Automated, high-throughput phenotyping. a. A coarse classification scheme for behavioral aging and death involving four states. b. The last few days of an individual’s life involve a set of stereotyped movement and morphological transitions. After a week or more of vigorous movement (purple), individuals slow down and exhibit only weak postural changes (yellow) until eventually ceasing all movement (red). Meanwhile, their apparent body size decreases until the time of death at which point it increases rapidly (green), plateaus, and then decreases again (gray). c. In a population of 368 wild-type animals, the times spent moving vigorously (pink), moving weakly (yellow), and non-moving (red), were annotated by hand and plotted. Each horizontal line represents a single individual, with individuals ordered vertically according to their death time. d. The same lifespan data is plotted with state occupancy times represented as fractions of lifespan, with individuals ordered vertically by the duration of vigorous movement. e. The performance of automated lifespan analysis using body size expansion as a marker for death. A Hidden Markov Model (HMM) was fit to 830 wild-type individuals housed in a variety of environmental conditions, with the population subdivided according to biological replicate and tested in a 6-fold cross-validation approach. By-hand annotated death times were compared to the death times identified by the HMM, shown both in respect to absolute time and f. as a cumulative distribution function (CDF) of the difference between by hand and automated measurements as a fraction of lifespan.

To quantify behavioral aging, we designed a classification scheme that divides an individual’s life into discrete behavioral and morphological periods: a “vigorous movement” period during which animals crawl freely across the plate, a “weak movement” period during which animals remain fixed at a single plate location while exhibiting repetitive head and body postural changes, an “alive but non-moving” period during which animals exhibit no detectable movement or postural changes (Fig 1A), and a “dead” state. This classification hews closely to the standard scheme developed for interpreting by-hand assays and other imaging methodologies [2, 4, 18]. The one additional period we add—describing living but non-moving individuals—arises because the lifespan machine, unlike previous methods, captures a morphological transition that occurs at the same time as death [28, 29] in which individuals exhibit a gradual decrease and then sudden increase in their apparent body size reflecting a type of rigor mortis or osmotic phenomena (Fig 1B). We find that death-time expansion is a robust marker of death across nematode taxa, observable in populations of *C*. *briggsae*, *C*. *tropicalis*, *C*. *japonica*, *C*. *brenneri*, and *P*. *pacificus* (S1 Text). Death-time expansion allows us to identify non-moving animals as alive until the time when they expand, and record them as dead afterwards. According to this scheme, we found that wild-type individuals at 20°C spent on average 64% of their lives moving vigorously, 32% of their lives moving weakly, and 3.6% of their lives non-moving (Fig 1C and 1D).

To rigorously and automatically segment individual aging trajectories according to this classification scheme, we developed a new version of the automated imaging platform. Combining Hidden Markov Models with other machine-learning approaches (S1 Text), we produced a system that we can train using by-hand annotated data sets and then employ to automatically classify behavioral and morphological states in novel image data (S1A Fig). We collected training on data from wild-type populations housed at a variety of temperatures and food sources, and found that our system could robustly identify death times with an average error of 10.9±0.7 hours (Fig 1E) or 1.4% of lifespan on novel, independent biological replicates (Fig 1F). In populations in which individuals varied in lifespan across a range of five weeks, we found 95% of individuals had their lifespan estimated within one day of their true lifespan, which corresponds to 95% of individuals estimated within 4.8% of their true lifespan (S1B and S1C Fig).

From these results, we conclude that our behavioral classification scheme and machine learning approach supports the measurement of lifespan at an accuracy and precision equivalent to or better than existing methods involving manual inspection of nematodes. Our new, “non-moving” behavioral state produces only a modest increase in apparent lifespan, with 30% of individuals spending no time non-moving and 50% of all individuals spending less than ten hours (S1D and S1E Fig) non-moving. However, we show that the morphological transition that marks the end of the non-moving period provides a practical, visible landmark for our HMM model, supporting the statistical accuracy and precision needed to jointly measure VMC times and lifespan at unprecedented statistical resolution and scale.

### The risk of VMC and death increase over time, influenced by shared heterogeneity

**Fig 2:**
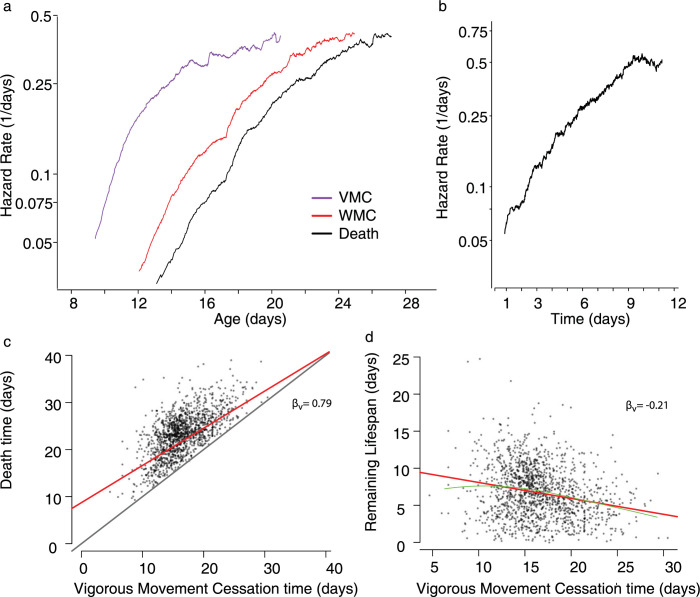
Relating vigorous movement and lifespan in wild-type populations. a. In a population of 1441 wild-type *C*. *elegans* housed at 20°C, the risk of animals vigorous movement cessation (VMC) (purple), weak movement cessation (WMC) (red), and the risk of death (black) are shown in respect to chronological age. b. The "clock-reset" hazard estimates the risk individual’s death as a function of the time spent alive after VMC. c. The relationship between each individual’s VMC and death times are shown, with the linear regression line (red) and unit line *y = x* (black) overlaid. d. The relationship between VMC and the lifespan remaining after VMC, with the linear regression line (red) and LOESS regression line (green) overlaid.

We first considered separately the temporal dynamics of VMC and death. For 1441 wild-type individuals housed at 20°C, we estimated the event-specific hazard rates describing the risk of ceasing vigorous movement and the risk of dying as both risks increase with age. We find that the cessation of vigorous movement and cessation of weak movement exhibited remarkably similar temporal dynamics to those previously observed for death [30–33], involving a rapid increase in rate starting midlife followed by a gradual deceleration later in life (Fig 2A). Similar dynamics were observed across all replicates collected, whose shape we characterize in detail using parametric fits in the supporting text S2 Text.

To consider the relationship between the hazard trajectories of VMC and death, we considered a well-known phenomenon in demographic aging: mortality deceleration. Age-associated increases in the risk of death have been observed to decelerate at old ages in nematode [33], fly [32], and humans [34]. Theory predicts that deceleration can arise in two ways: 1) a physiologic deceleration in each individuals’ rate of aging or, alternatively, 2) a compositional phenomenon in which fixed population heterogeneity in the aging rate causes fast-aging subpopulations to die early, leaving only slow-aging subpopulations alive at late ages [9, 34, 35]. We observe late-life deceleration in the risk of both VMC and death (Fig 2A), which is consistent with either explanation for mortality deceleration.

We then considered the risk of death specifically in the time following VMC—estimating the “clock-reset” hazard rate [10, 36] (statistical methods) in which the population hazard rate is calculated as a function of the time that has passed since each individuals’ VMC, rather than as a function of chronological time. We find that after roughly one day after ceasing vigorous movement—the first time at which enough deaths occur for us to accurately estimate the hazard rate—individuals experience a steady increase in their risk of death with much less mortality deceleration in respect to a baseline Weibull hazard function (Fig 2B and S2 Text) compared to chronological-time hazard function (Fig 2A). This finding allows us to differentiate between the two theoretic origins proposed for mortality deceleration in *C*. *elegans* described above. Because the risk of death continues to increase after VMC, even accounting for variation in VMC time across the population, we learn that the aging process determining death times continues after vigorous movement ceases. Yet, because mortality deceleration is greatly attenuated in the “clock-reset” hazard relative to the chronological-time hazard functions, we learn that a substantial part of population heterogeneity influencing death times must already be fixed at the time of VMC, and that this heterogeneity must produce most of the mortality deceleration observed in the chronological-time hazard functions. Therefore, our data identifies population heterogeneity as the largest source of mortality deceleration in *C*. *elegans* death hazard functions. We further conclude that the hazard trajectories of VMC and death have similar shapes because they are both determined by one or more aging processes that influenced by some shared source of physiologic heterogeneity that links variability in VMC to deceleration of the death hazard function.

We then considered the “clock-reset” hazard function describing the remaining lifespan after weak movement cessation (WMC). We find this span exhibits dramatically different temporal dynamics compared to VMC: a large fraction of the population spend less than two hours non-moving before death, leading to an initially high hazard followed by a four-day period involving only a two-fold increase in the risk of death—much smaller than the 10-fold increase observed for VMC (Fig 2B). The small increase in the risk of death following WMC suggests that this period does not describe a process with kinetics distinct from VMC and lifespan. Though the period of non-movement is important for accurate identification of death times, its relatively flat clock-reset hazard function suggests that it does not necessarily demarcate a uniquely informative step in the aging process. Therefore, for the purposes of further analysis we consider the non-moving but alive period as an extension of the weak moving period, defining a span beginning at VMC and ending at death—a span showing an unambiguous aging kinetics (Fig 2B).

### VMC times and lifespan are related by an additive, linear model with a slope less than one

We then set out to study the correlation between VMC time and lifespan. Considering the same population as before, in agreement with previous studies [2, 4, 17, 18, 27] we find that VMC and death times are strongly correlated across individuals (Fig 2C) (partial R^2^ = 0.53, “statistical methods”). Previous studies have related VMC and lifespan in two contrasting ways, either using a proportional model in which VMC is quantified as a fraction of lifespan—e.g “individuals spend 64% of their lives moving vigorously”—or using an additive model in which VMC occurs at an additive time before death—e.g “Individuals remained alive for 9 days after VMC”. We reasoned that our larger, more resolved data set should allow us to compare these additive and proportional models by quantifying the heteroskedacity of residuals derived from regression models assuming either an additive or proportional relationship. We find that VMC times and lifespan are substantially better described by the linear, additive model (White test score: 28; p = .015) compared to a linear, proportional model (White test score: 233; p < 10^-10) (S2B and S2C Fig). Therefore, we conclude that our data suggests that vigorous movement is not best described as a proportional period of lifespan, and VMCs are not best represented as fractions of lifespan. Instead, VMC and death describe distinct spans better described in terms of absolute time, whose durations do correlate but not because one inherently arises in proportion to the other.

We then estimated β_v_, the slope of the linear additive model. We find that the slope is significantly less than one (β_v_ = 0.79 95% CI (0.708,0.872); bootstrap p(β_v_ < 1) < 10^-10). Such a slope suggests that individuals who remain vigorous longer than their peers subsequently spend less time remaining alive than their peers, an effect that can be shown directly as an inverse correlation between the vigorous movement period and remaining lifespan (Fig 2D) (β_v_ = -0.21; p (β_v_! = 0) < 10^-10).

To evaluate the robustness of this inverse correlation across experimental replicates, we assembled a larger data set of time-lapse imagery describing the lives and deaths of 7861 wild-type individuals, representing a population approximately thirty times larger than any previous similar study [4, 17, 23, 27]. Our data contains ten biological replicates with on average 810 individuals each, with replicates consisting of both new and previously published [28, 33] experiments. To ensure our findings were robust across diverse environmental conditions, we included populations housed at either 20°C or 25°C and fed either live or UV-inactivated *E*. *coli*. In this data, we observe a linear relationship between vigorous movement and lifespan with a slope less than one in seven out of ten replicates (S2D and S2F Fig). The estimated slope depended neither on environmental temperature nor food source (S2F Fig), and could not be explained by measurement error in our apparatus (S4 Text). The estimated slope did not derive from any truncating effect of death on VMC times, as almost all individuals cease vigorous movement several days before death. Instead, our slope estimates vary between replicates at a value centered on 0.91. Therefore, we attribute the variation in slope between replicates to some uncontrolled environmental factor inherent to the standard nematode culture protocol, the existence of which might explain contradictory quantitative findings in previous studies relating [2, 17, 23, 27] behavioral aging rates to lifespan, as previous efforts have included only one or a small number of replicates.

In summary, we find that VMC times and lifespan share a linear, additive relationship, whose slope has a value significantly less than one. Our analyses additionally highlight the importance of performing multiple, large-population-size replicate when relating behavior to lifespan, as these replicates are necessary to control for the influence of batch effects.

### The negative correlation between VMC times and remaining lifespan is robust across mutations, interventions and timescales

**Fig 3:**
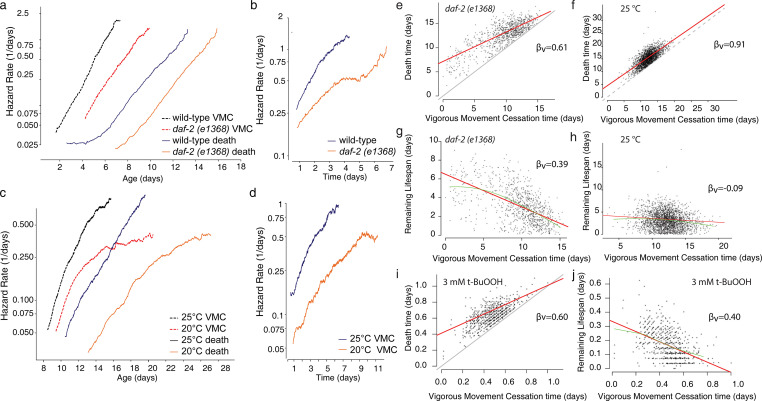
The effect of interventions on vigorous movement and lifespan. a. In a population of 919 wild-type (purple) and 906 *daf-2(e1368)* (black) individuals, we estimated the risk of vigorous movement cessation (VMC) (solid) and the risk of death (dashed). b. In the same population, we estimated the "clock-reset" risk of an individual’s death after VMC for wild-type populations (light red) and *daf-2(e1368)* populations (dark red). c. For 1441 wild-type individuals housed at 20°C (black) and 2346 individuals housed at 25°C (purple), we estimated the same risks, for VMC (solid) and death (dashed). d. the “clock-reset” risk of death for the same populations as c. e. The relationship between each individual’s death and VMC times in a *daf-2(e1368)* population, with the linear regression line (red) and unit line *y = x* (black) overlaid. f. The same analysis but for a wild-type population housed at 25°C. g. The relationship between VMC times and lifespan remaining after VMC for the *daf-2(e1368)* population, with the linear regression line (red) and LOESS regression line (green) overlaid. h. The same analysis but for wild-type animals housed at 25°C. i. The relationship between VMC and lifespan for wild-type populations exposed to 3 mM t-BuOOH j. The relationship between VMC and remaining lifespan for the same population.

Previous studies have identified several genetic factors that alter the relative duration of vigorous movement and lifespan [4, 17, 23, 24], while preserving the correlation between the two [4]. To study this in more detail, we first considered a mutant population expressing a variant of the insulin/IGF receptor, *daf-2 (e1368)* known to extend youthful vigor and lifespan [4, 17, 23, 24]. The *e1368* allele was chosen as having fewer “type II” side effects on development and behavior compared to *e1370* while nevertheless producing substantial lifespan extension. We find that *daf-2 (e1368)* dramatically slows age-associated increases in the risk of both VMC and death (Fig 3A). *daf-2 (e1368)* also slows increases in the “clock-reset” risk of death during the period after VMC (Fig 3B). Considering the effect of changes in environmental temperature in wild-type populations, we found that populations housed at 20°C, compared to 25°C, exhibited a delayed increase in the risk of VMC similar to *daf-2 (e1368)*, slowing the age-associated increases in both hazard functions (Fig 3C and 3D).

We then asked whether either *daf-2(e1368)* or changes in environmental temperature would alter the correlation between VMC times and lifespan. We found that all populations exhibited a linear relationship with a slope of less than one (Fig 3E–3h), suggesting that mutations and interventions can alter population-average VMC and death times while preserving the correlation between VMC timing, lifespan, and remaining lifespan between individuals. To further probe the plasticity of this inverse correlation, we then considered a third, lifespan-altering intervention: exposure to tert-butyl hydroperoxide (t-BuOOH), an environmental source of oxidative stress that dramatically shortens lifespan. We found that populations whose lifespan was shortened by t-BuOOH almost ten-fold nevertheless exhibited a linear relationship between vigorous movement and lifespan, at both 1.5 mM and 3 mM, with a slope less than one at each concentration (Figs 3I and 3J, S3A and S3B): 3 mM: β_v_ = 0.60, p (β_v_ < 1) < .0001, 1.5 mM: β_v_ = 0.85, p (β_v_ < 1) = 0.014, Taken together, our data describing *daf-2*, temperature, and t-BuOOH show that the correlation between VMC times and lifespan is not specific to any particular timescale, and is a property of *C*. *elegans* aging and death regardless of whether this occurs over the course of several weeks, several days, or overnight.

Finally, we expanded our analysis to include five additional mutations and interventions drawn from new and previously published [28] datasets, including mutations and interventions both with known effects on VMC and lifespan and those not yet characterized. Because alterations in mitochondrial function have been shown to disproportionately affect behavior and lifespan [23], we characterized populations sharing the *nuo-6(qm200)* allele, which extends lifespan by disrupting mitochondrial complex I function [25]. We characterized populations exposed to Antimycin A which induces paralysis via depolarizing mitochondrial membrane potential and in small doses has been shown to extend lifespan [37]. To induce paralysis without directly effecting a pathway known to influence lifespan, we characterized a population with the *unc-119(ed3)* allele, which disrupts neuronal morphogenesis to produce a profound paralysis [38]. We considered three dietary changes known to extend lifespan, characterizing animals fed ultraviolet-light-inactivated *E*. *coli* [22], a population fed live bacteria but then deprived of all food starting on the second day of adulthood, and finally a population sharing the *eat-2(ad1116)* allele which extends lifespan by inhibiting feeding [39]. Finally, we considered populations sharing the *glp-1(e2141)* allele which extends lifespan by ablating the germline [40].

We observed a linear relationship between VMC and death times with a slope less than one in *nuo-6 (qm200)* populations, in *unc-119 (ed3)* populations, and in populations exposed to UV-treated bacteria (Table 1 and S3C Fig). In populations exposed to Antimycin A, most individuals exhibited a very short vigorous movement period making it impossible to precisely resolve inter-individual differences in VMC times and correlate them to lifespan. *glp-1 (e2141)* did not exhibit an inverse relationship between vigorous movement and remaining lifespan, but neither did the wild-type control for that experiment. We found that *eat-2 (ad1116)* attenuates the inverse relationship between vigorous movement and remaining lifespan, unique in this regard. We confirmed this attenuation in a second replicate *eat-2(ad1116)* populations, and additionally observed the same attenuation though to a lesser magnitude, in wild-type animals deprived of all food starting on day 2 of adulthood (S3D and S3E Fig).

**Table 1:** The effect of interventions and mutations on the slope of the linear model relating VMC and death times. Parameter estimates of βv and the p-value testing the hypothesis (1-βv)! = 0. Confidence intervals and p-values were obtained via bootstrapping.

Intervention	N	β_v_	β_i_ 95% CI	(1-β_i_)	(1-β_i_) 95% CI	p value β_i_ ⩵ 1	Temperature	Diet
*eat-2 (ad1116) R2*	737	1.03	(0.874,1.192)	0.03	(-0.187, 0.109)	0.68	20	Live
Bacterial Deprivation	757	0.91	(0.775,1.038)	-0.09	(0.012, 0.170)	0.01	20	Live
wild type	991	0.87	(0.764,0.980)	-0.13	(0.047, 0.208)	0.0015	20	Live
wild type	544	0.79	(0.698,0.891)	-0.21	(0.122, 0.295)	< 1e-10	25	HT115
*eat-2 (ad1116) R1*	578	1.17	(0.968,1.368)	0.17	(0.122, 0.295)	0.99	20	Live
wild type	1239	0.64	(0.526,0.754)	-0.36	(0.242, 0.481)	< 1e-10	20	Live
wild type	205	1.15	(1.009,1.300)	0.15	(-0.252,-0.044)	1	25	Live
*glp-1(e2141)*	214	0.92	(0.786,1.061)	-0.08	(-0.060, 0.233)	0.16	25	Live
9.3 uM Antimycin	308	1.21	(0.643,1.770)	0.21	(-0.796, 0.366)	0.73	20	UV
4.3 uM Antimycin	276	1.03	(0.586,1.468)	0.03	(-0.460, 0.436)	0.55	20	UV
5.6 uM Antimycin	312	0.91	(0.488,1.331)	-0.09	(-0.426, 0.625)	0.36	20	UV
0 uM Antimycin	361	0.2	(-0.091,0.483)	-0.8	(0.330, 1.143)	< 1e-10	20	UV
UV-Inactivated	615	0.8	(0.671,0.930)	-0.2	(0.024, 0.374)	0.015	20	UV
Live	1239	0.64	(0.526,0.754)	-0.36	(0.248, 0.485)	< 1e-10	20	Live
25 C	2346	0.91	(0.847,0.977)	-0.09	(0.027, 0.149)	0.0032	25	UV
20 C	1441	0.79	(0.708,0.872)	-0.21	(0.130, 0.284)	< 1e-10	20	UV
0 mM t-BuOOH	1409	1.01	(0.965,1.062)	0.01	(-0.065, 0.039)	0.7	25	Live
1.5 mM t-BuOOH	848	0.85	(0.709,0.995)	-0.15	(0.015, 0.234)	0.014	20	Live
6 mM t-BuOOH	741	0.67	(0.331,1.015)	-0.33	(0.044, 0.679)	0.0072	20	Live
3 mM t-BuOOH	706	0.6	(0.405,0.789)	-0.4	(0.122, 0.829)	< 1e-10	20	Live
*nuo-6 (qm200)*	133	0.77	(0.679,0.857)	-0.23	(0.123, 0.351)	< 1e-10	20	UV
wild type	328	0.7	(0.523,0.881)	-0.3	(0.127, 0.437)	0.0008	20	UV
wild type	912	0.83	(0.694,0.964)	-0.17	(0.055, 0.303)	0.0036	25	Live
*daf-2 (e1368)*	898	0.61	(0.519,0.703)	-0.39	(0.269, 0.526)	< 1e-10	25	Live
wild type	99	1.08	(0.877,1.290)	0.08	(-0.254, 0.120)	0.8	25	Live
*unc-119 (ed3)*	181	0.72	(0.470,0.974)	-0.28	(0.031, 0.520)	0.014	25	Live

In summary, we conclude that many lifespan-altering mutations and interventions can dramatically alter the timing of both VMC and death while preserving the correlation between VMC and death times and, furthermore, the inverse relationship between VMC and remaining death times. This correlation and negative relationship does not depend on any particular relative timescale of VMC or lifespan, as we see it in populations aging over the course of two weeks and populations aging over the course of twenty-four hours. Instead, the correlation and negative relationship between VMC and remaining death time appears to reflect some structural aspect of aging that remains consistent across many timescales, mutations, and interventions.

### VMC and death times are determined by distinct aging processes

Our results highlight a phenomenon that demands explanation: why do many aspects of the quantitative relationship between VMC and lifespan remain robust across so many timescales and genotypes? Previous work has interpreted a correlation between VMC timing and lifespan as suggesting that some upstream mechanism links their timing [4]. So, we set out to understand how our new measurements of this correlation inform us about the physical decline or declines determining VMC timing and lifespan.

To start, we focused on the inverse correlation between VMC timing and remaining lifespan. This correlation reflects a pattern present in our data in which all individuals, immediately after ceasing vigorous movement, do not share the same expected remaining lifespan (Figs 2D, 3G, 3H and 3J). Instead, an individual’s remaining lifespan after VMC decreases according to the chronological time at which VMC occurs. Therefore, individuals with early and late VMC times cannot be physiologically identical at the time of their VMC, as these individuals must differ according to some physiological state responsible for their differing in remaining lifespan. In consequence, individuals must have at least two physiologic states corresponding to distinct aging processes: one determining their VMC time and another determining their death time. Thus, from the robustness of the observed correlation and slope between VMC times and lifespan we can draw a remarkable conclusion that individual *C*. *elegans* must have at least two distinct “biological ages”: one biological age determining the timing of VMC the other determining the timing of death.

To formalize and justify our reasoning, we adopt quantitative methods drawn from stochastic process theory and in particular, the field of Markov processes. Markov processes—the most accessible and familiar example being “Brownian motion”—are mathematical models that describe the behavior of any system whose future behavior depends only on its current state. Markov processes are useful tools for understanding aging phenomena [9, 10] in part because they capture the intuitive assumption that future outcomes in an individuals’ life depend on their current physical state (a “biological age”) not some external clock. In this way, any model that describes a “biological age” as distinct from “chronological age” is likely implicitly assuming a Markovian aging process. Markov processes have been used to describe aging processes previously [9, 10], but here we for the first time apply them to rigorously model the causal relationship between VMC and lifespan.

**Fig 4:**
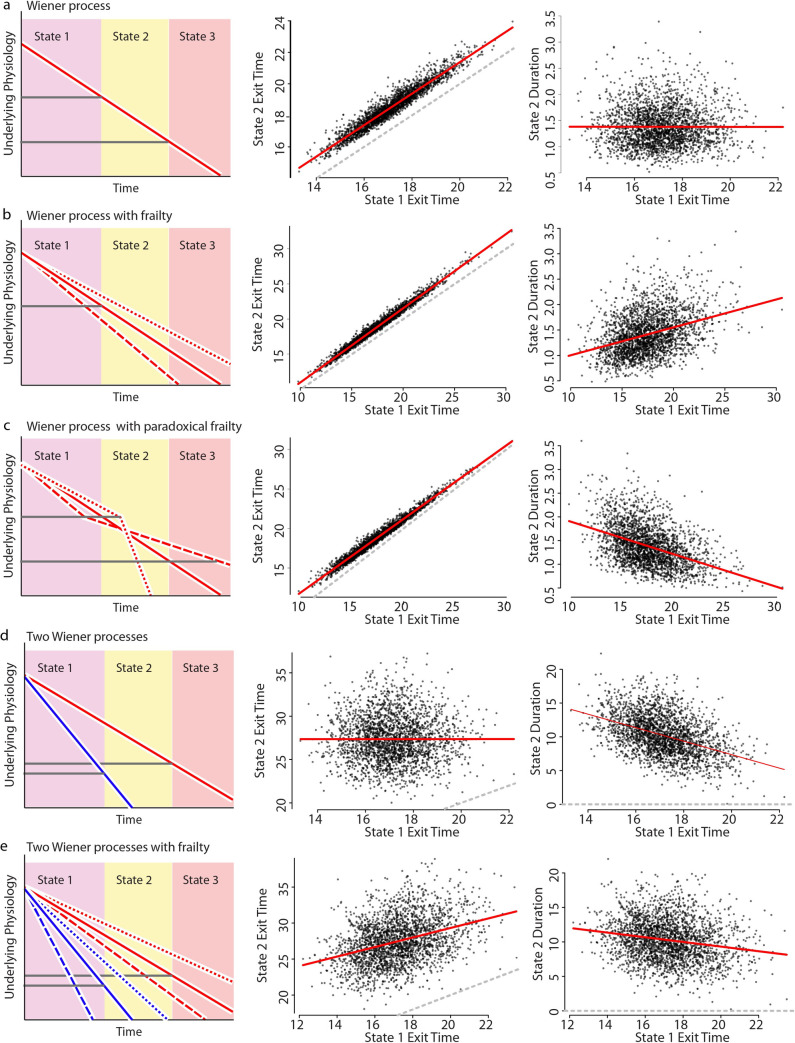
Stochastic models of aging link VMC and death times. To explain our experimental data, we performed a series of stochastic simulations involving one or two biased random walks (Weiner processes; see *statistical methods*). The simulation generates transition times for a set of individuals between three states, with state 1 exit corresponding to the vigorous movement cessation (VMC) observed experimentally and state 2 exit corresponding to death. Each row explores a different set of assumptions about the relationship between these state transitions, as diagrammed in the left column. Simulation results are summarized by comparing the timing of each individual’s exit from states 1 and 2 (middle column) with the linear regression (red) and *x = y* unity lines overlaid. Simulation results are further summarized by comparing each individuals’ state 1 exit time to duration they spent in state 2 (right column), with the linear regression line overlaid (red). This latter duration corresponds to the lifespan remaining after VMC as observed experimentally. a. A single process model in which state transition times are determined by the sequential crossing of two thresholds. b. A single process model as in *a*., but including population-heterogeneity (frailty) in individual aging rates. c. A single process model as in *b*., but including a paradoxical frailty effect in which the effect of population-heterogeneity instantaneously flips between states, such that the relative rate of aging is reversed between individuals when they exit state 1. d. A two process model in which the exit from states 1 and 2 are determined by distinct processes (red and blue). e. A two-process model like *d*. but including population-heterogeneity (frailty) in individual aging rates.

We approached the question both analytically (S3 Text) and through simulation (Fig 4A). We found that a single-state Markovian process is generally unable to produce the observed linear relationship between VMC and death times with a slope less than one. Were VMC and death times determined by a single Markov process, individuals would necessarily share equivalent biological ages at the time of VMC. In consequence, individuals with equivalent biological ages would share equivalent expected remaining lifespan after VMC, regardless of the chronological time that VMC occurred which is in conflict with our data. We could only identify one approach for reconciling our data with a single-state Markov model, which involved artificially inverting the relative rate of aging between individuals before and after the time of VMC. If individuals aging more slowly than their peers early in adulthood instantly switch to aging faster than their peers at the time of their VMC, then the resulting correlation between VMC and death times are reconciled with our data (Fig 4C). However, we are unaware of a mechanism that could explain such an inversion of the relative aging rate, and in *C*. *elegans* [41], rodents [42] and humans [43] increases in vigorous movement extend lifespan. Therefore, we conclude that VMC and death cannot be explained as resulting from a single physical decline following single-state Markovian dynamics.

We then explored the behavior of an aging system described by a two-state Markov process. We found that two fully independent states failed to replicate the correlation between VMC and death times (Fig 4D), as the correlation between VMC and death times requires that the two states must be somehow non-independent. Still, the two states cannot be completely non-independent or else the slope of the linear, additive relationship would be exactly one and therefore conflict with our data (S3 Text). So we explored the intermediate model in which two Markov processes are partially independent. The simplest model for this involved two Weiner processes whose average rates are influenced by a shared upstream factor (Fig 4E). We call such a system a “hierarchical process model” because two Markov processes encode distinct states whose average rate of change depends on a shared upstream factor. Using simulations (Figs 4E and S4) and analytic calculation (S3 Text), we demonstrate how such a hierarchical organization of a two-process system will generically produce correlations that match our empiric data, with the linear relationship between VMC and death times showing a slope less than one.

Taken together, our modeling and simulations clarify the constraints that our measurements place on the causal relationship between VMC and death. Our data requires that *C*. *elegans* has at least two distinct but co-varying physical declines, summarized by at least two separate “biological ages”. This model is consistent with another formal study of aging processes, performed in mice, that found the dynamics of cellular senescence and death well-described by two distinct but co-varying physical declines [44].

### Environmental factors, diet, and mutations act both proportionally and disproportionally to alter behavioral aging and lifespan

**Fig 5:**
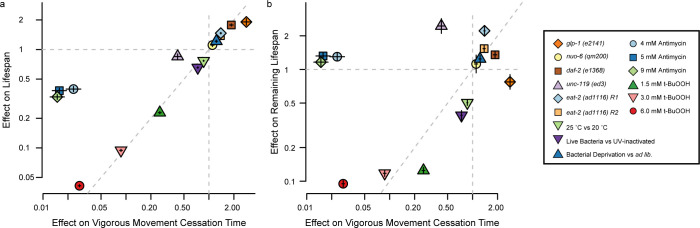
The relative effects of mutations and interventions on vigorous movement and lifespan. a. The effect of 15 interventions on vigorous movement, lifespan, and remaining lifespan are shown, with each point showing an interventions’ effect on VMC (x-axis) and lifespan (y-axis) or b. remaining lifespan (y-axis). Intervention effects are quantified as the fold-change in VMC times, death times, or remaining lifespan observed in the treated population relative to untreated controls (usually wild-type animals at 20°C). The interventions tested were *glp1(e2141)* (orange diamond), *nuo-6(qm200)* (yellow circle), *daf-2(e1368)* (brown square), *unc-119(ed3)* (light purple triangle), two replicates of *eat-2(ad1116)*: (blue diamond and orange square), the effect of exposure 25°C throughout adulthood compared to the same period spent at 20°C (green inverted triangle), the effect of a diet consisting of live *E*. *coli* compared to a diet of UV-inactivated E. coli (purple inverted triangle), bacterial deprivation (blue triangle), 4, 5, and 9 mM Antimycin A, (light blue circle, blue square, green diamond), and 1.5 mm, 3mM and 6 mM t-BuOOH (green triangle, pink triangle, red circle). Error bars indicate bootstrapped 95% confidence intervals.

The hierarchical process model requires that three distinct aspects of physiology exist within each individual: a physical decline that determines the timing of VMC, a physical decline that determines the timing of death, and a shared upstream factor that influences the rate of both. So, we then asked whether such a model could explain why some mutations and interventions have been characterized as “uncoupling” of VMC times and lifespan [17, 23, 24]. Considering a set of nine mutations and environmental interventions previously characterized as influencing lifespan, we compared the effects of each intervention on the typical time spent moving vigorously and lifespan. Because VMC times and lifespan are related by a linear, additive model, we applied accelerated failure time (AFT) regression model to separately quantify each intervention’s influence on VMC and lifespan (Fig 5A). Using this model, we compared each mutant or intervention relative to the appropriate control, in most cases a temperature-matched wild-type population. We found that, with the notable exception of paralytic agents Antimycin A and *unc-119(ed3)*, mutations interventions followed a general trend for proportionate effects on VMC and death times, evidenced by their alignment across a twenty-fold change in timescale along the diagonal in Fig 5A. Therefore, we conclude that at a course granularity, most interventions in aging do not appear to “uncouple” VMC times and lifespan. Across several orders of magnitude of effects, mutations and interventions that shorten lifespan tend to decrease VMC times by approximately the same extent, and vice versa (S5A Fig and S5 Text)

At a fine granularity, however, our data confirms previous work that identifies mutations and interventions as producing statistically significant disproportionate effects on VMC times and lifespan. These disproportional effects are highlighted by the comparison between VMC times and each individual’s remaining lifespan after VMC (Fig 5B). We observed mutations and interventions can produce all possible combinations of effects—delaying VMC while increasing remaining lifespan, delaying VMC while decreasing remaining lifespan, hastening VMC while shortening remaining lifespan, and hastening VMC while shortening remaining lifespan (Table 2). It is important to note that these disproportionate effects (Fig 5B) exist mostly as small deviations from the overall proportional trend (Fig 5A). We therefore propose a two-grain model to describe the effect of interventions in aging on VMC timing and lifespan. At a coarse granularity, most mutations and interventions that produce large effects on VMC times produce similarly large effects on death, and vice versa. At a fine granularity, interventions do produce measurable disproportionate effects on VMC and death times.

**Table 2:** The effect of interventions and mutations on VMC, lifespan, and lifespan remaining after VMC. β_i_ is estimated effect size of each intervention on the three different periods of life, estimated using an Accelerated Failure Model (Statistical Methods). Cells are colored according to the effect size, blue indicating a lengthening of the period of life and red indicating a shortening.

	VMC			Lifespan			Remaining lifespan after VMC		
Intervention	β_i_	β_i_ 95% CI	p	β_i_	β_i_ 95% CI	p	β_i_	β_i_ 95% CI	p
*glp-1(e2141)*	2.84	(2.5,3.2)	< 1e-10	1.94	(1.8,2.1)	< 1e-10	0.8	(0.7,0.9)	4.30E-03
*daf-2 (e1368)*	1.87	(1.8,2.0)	< 1e-10	1.78	(1.7,1.8)	< 1e-10	1.36	(1.3,1.5)	< 1e-10
*eat-2 (ad1116) R2*	1.36	(1.3,1.4)	< 1e-10	1.42	(1.4,1.4)	< 1e-10	1.9	(1.7,2.1)	< 1e-10
*eat-2 (ad1116) R2*	1.38	(1.4,1.4)	< 1e-10	1.54	(1.4,1.66)	< 1e-10	1.38	(1.35,1.42)	< 1e-10
Bacterial Deprivation	1.23	(1.2,1.27)	< 1e-10	1.24	(1.15,1.34)	6.20E-08	1.241	(1.15,1.34)	6.20E-08
*nuo-6 (qm200)*	1.11	(1.0,1.2)	5.20E-04	1.17	(1.1,1.2)	< 1e-10	1.71	(1.4,2.2)	7.00E-06
25 C	0.86	(0.9,0.9)	< 1e-10	0.77	(0.8,0.8)	< 1e-10	0.5	(0.5,0.5)	< 1e-10
Live Bacteria vs UV	0.72	(0.7,0.7)	< 1e-10	0.64	(0.6,0.7)	< 1e-10	0.35	(0.3,0.4)	< 1e-10
*unc-119 (ed3)*	0.42	(0.4,0.5)	< 1e-10	0.85	(0.8,0.9)	2.40E-07	2.45	(2.1,2.8)	< 1e-10
1.5 mM t-BuOOH	0.26	(0.2,0.3)	< 1e-10	0.23	(0.2,0.2)	< 1e-10	0.13	(0.12,0.13)	< 1e-10
3 mM t-BuOOH	0.087	(0.084,0.090)	< 1e-10	0.094	(0.092,0.097)	< 1e-10	0.12	(0.11,0.13)	< 1e-10
6 mM t-BuOOH	0.028	(0.027,0.029)	< 1e-10	0.041	(0.040,0.042)	< 1e-10	0.095	(0.09,0.10)	< 1e-10
4.3 uM Antimycin	0.024	(0.019,0.031)	< 1e-10	0.42	(0.4,0.4)	< 1e-10	1.34	(1.2,1.5)	4.50E-09
9.3 uM Antimycin	0.017	(0.011,0.025)	< 1e-10	0.35	(0.31,0.40)	< 1e-10	1.14	(1.0,1.3)	1.10E-01
5.6 uM Antimycin	0.013	(0.010,0.017)	< 1e-10	0.35	(0.32,0.38)	< 1e-10	1.26	(1.1,1.4)	5.50E-06

We then considered how interventions could be acting at two granularities to act both proportionately and disproportionately at different scales, and realized that two granularities emerge naturally from the hierarchical process model. The coarse-grained, proportional effects will arise when interventions alter the rate of two aging processes equally, accomplished indirectly by some action on the upstream factor in our hierarchical process model. The fine-grained, disproportional effects will arise when interventions act directly on a specific aging process, influencing one process’s rate more than the other. In this way, we find that the hierarchical process model not only explains the observed correlation structure between VMC times and lifespan within each populations, but additionally explains the two-grain, proportional and disproportional effects of interventions between populations.

### Single interventions can act both proportionately and disproportionally to alter VMC timing and lifespan

So far, our interventional study of VMC and lifespan involved comparisons between diverse interventions that act via diverse mechanisms to alter VMC timing and lifespan. To study the relationship between VMC timing and lifespan in a more controlled setting we sought to develop an experimental system in which the dose-response relationship between a single molecular mechanism, VMC times, and lifespan could be measured. To realize this goal, we used the auxin-inducible degradation (AID) system [45] to obtain long-term, tunable *in vivo* control of VMC timing and lifespan. We focused on a central mechanism of molecular and cellular biology, transcription, reasoning that because transcription is required for normal lifespan (S6A Fig) the quantitative disruption of RNA polymerase II might produce a quantitative shortening of lifespan that we could compare to its effect on VMC times. Editing the endogenous locus of RNA Polymerase II complex subunit *rpb-2* to introduce a short degron tag [46], we obtained a strain that allowed us to selectively ubiquitinate and degrade RPB-2 using the auxin-inducible *Arabidopsis* TIR1 E3-ubiquitin ligase transgene. We hypothesized that chronic exposure to different auxin dosages would produce differences in the steady-state degradation rate of *RPB-2*::*AID* and thus yield different average activity levels of the RNA Polymerase II complex. By exposing populations to a dosage series of auxin, we predicted that we could obtain a dose-response curve linking quantitative changes in *RPB-2* activity to its quantitative influence on both VMC timing and lifespan.

**Fig 6:**
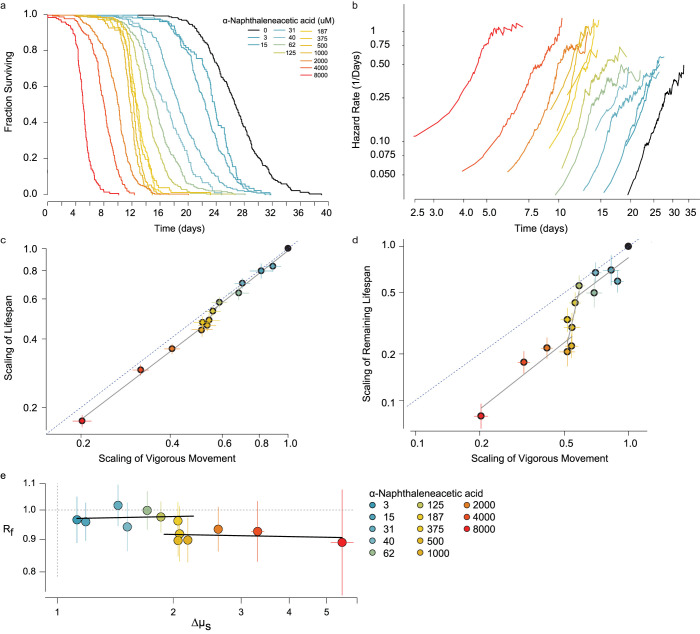
An RNA Polymerase II dosage series. Tunable, *in vivo* manipulation of RNA polymerase II activity using an *rpb-2* auxin-inducible degron. A. The lifespan of *rpb-2*::*AID;TIR1* populations exposed to a set of increasing concentrations of alpha-Naphthaleneacetic acid (NAA), shown as Kaplan-Meier survival curves. b. The time-dependent trajectory of the risk of death calculated for the same populations. c. At each dosage, the effect of NAA on the time of vigorous movement cessation and lifespan are compared. Error bars indicate 95% confidence intervals of AFT regression parameter estimates. Data was fit by a tri-phasic linear model (grey lines; statistical methods). Dotted line indicates the *x =* y unity line. d. The same analysis, but comparing vigorous movement cessation to remaining lifespan. e. Estimates of R_f_ and Δμ, quantifying the magnitude of disproportionate and proportionate effects of each NA concentration on VMC and lifespan (S5 text; Statistical methods). Error bars indicate 95% confidence intervals. Black lines indicate linear fits to each concentration range; < 187 μM and > 375 μM.

We obtained a transgenic line *rpb-2(cer135)* in which the endogenous *rpb-2* locus was edited to include a GFP::AID::3xFLAG tag, which we combined with a *peft-3*::*TIR1* ubiquitin ligase. We observed that the auxin analog ɑ-Naphthaleneacetic acid (NA) [47] produces a dose-dependent, monotonic decrease in the lifespan (Fig 6A). At 8 mM NA, a relatively high dosage, we found that *rpb-2*::*AID* animals lived on average 81% shorter compared to unexposed animals. In contrast, control animals lacking *rpb-2*::*AID* and TIR1 lived only 16% shorter, indicating a small degron-independent effect of NA on lifespan.

We then considered the effect of RPB-2 degradation on each population’s transition-specific hazard function. We found that NA exposure increased the rate of increase in the risk of animals ceasing vigorous movement as well as the rate of increase in their risk of death (Fig 6B) in the same manner as we observed resulting from mutation of *daf-2(e1368)* and changes to temperature (Fig 3A and 3C).

We then considered the dose-response relationship between NA treatment, VMC timing and lifespan. We found a triphasic relationship (Fig 6C and 6D) well-represented by three piecewise linear regimes. At concentrations below 187 μM, RPB-2 degradation produced a disproportionately greater effect on VMC times than on lifespan, with lifespan and VMC times locked in proportion as NA altered both. Between 187 μM and 500 μM, we found that increasing dosages of NA produced a negligible effect on the vigorous movement stage but progressively shortened lifespan. Finally, at concentrations higher than 500 mM NA, RPB-2 degradation again produced a disproportional effect on vigorous movement and lifespan, with lifespan and VMC times locked in a constant ratio—a different ratio than that observed below 125 μM—as NA altered both lifespan and VMC times. To precisely estimate these ratios, we developed an AFT regression model that quantifies two effects of an intervention: the ratio R_f_ that describes the disproportional effect of NA on VMC and death times and the scale factor Δμ_s_ that describes the proportional effect of NA on VMC and death times (S5 Text). Using this model, we found that R_f_ does not change consistently with NA below 187 μM and above 500 μM, even NA progressively increases Δμ_s_ across these ranges. Together, these results demonstrate that NA produces a tri-phasic dose-response curve in respect to its effects on VMC and death times.

To better understand this tri-phasic dose-response curve, we compared the effects of NA on our *rpb-2*::*AID; TIR1* strain to the effects of NA on a control *rpb-2(+); TIR1* strain lacking the *rpb-2* degron tag. In a second replicate, we confirmed the distinct NA dose regimes of the *rpb-2*::*AID; TIR1* strain and demonstrated that the *rpb-2(+);TIR1* strain exhibited a much smaller (20%) shortening of lifespan at 8mM NA compared to the 600% shortening in the *rpb-2*::*AID; TIR1* strain (S6B and S6C Fig). However, across the range of 0 to 8mM NA, we observed NA to produce similar disproportionate effect on VMC and death times in both the *rpb-2*::*AID; TIR1* and *rpb-2(+);TIR1* strains. To quantify this effect, we again fit the proportionate/disproportionate AFT model and found that both strains exhibited similar relationships between R_f_ to NA, suggesting that NA’s effect on R_f_ acts through a mechanism independent of rpb-2::AID and distinct from the determinants of Δμ_s_. We therefore interpret the segmental dose-response as follows: Across the whole dosage regime, NA directly modulates RNA Polymerase II activity via the degron to produce proportional effects of vigorous movement and lifespan, which we observe as changes to Δμ_s_. However, at concentrations above 125 μM, NA acts independently of the RNA Polymerase II degron to shorten lifespan without effecting VMC, thereby acting disproportionally on VMC and lifespan and decreasing R_f_. These *rpb-2*::*AID* dependent and independent effects of NA combine to create two regimes in which lifespan and VMC change proportionately under the influence of *rpb-2* but delineated by the different proportion in each regime resulting from influence of *rpb-2*::*AID*-independent NA effects. This interplay between *rpb-2*::*AID*-dependent and independent mechanisms on VMC and death times supports our model of a hierarchical process model of VMC timing and lifespan—with NA influencing both VMC times and lifespan via *rpb-2*::*AID*’s influence on a shared factor upstream of both aging processes determining VMC and death times, but with NA also acting independently of *rbp-2*::*AID* to influence only the aging process determining death times.

## Discussion

In this study, we describe an automated imaging platform that we apply to explore the relationship between vigorous movement cessation (VMC), death, and the underlying physical declines that determine their timing. Previous work has shown that individuals that cease VMC earliest experience a disproportionately long “twilight” of late-life “decrepitude” [2], leading to a negative relationship between VMC and remaining lifespan. We show that this negative slope is preserved by most mutations and interventions in aging, even while such mutations and interventions produce large, disproportionate changes to population-average VMC and death times. We find that the inverse relationship between VMC and remaining lifespan is preserved in populations dying over the course of one, ten, or twenty days, suggesting it reflects some aspect of biological organization invariant across this wide range of timescales.

Through analytic calculation and simulation, we demonstrate how the additive, linear relationship between VMC timing and its stability across mutations and interventions is generic behavior of any system in which at least two distinct physical declines progress under the influence of a shared upstream factor. Formally, this means that the timing of VMC and death cannot be determined by a single Markov process, instead requiring that at least two distinct time-dependent states exist. Our results place the common admonition to students that “correlation does not equal causation” on firm empiric footing—the correlations we observe between VMC times and lifespan appear to be the result of the “confounding” effect of systemic factors that cause the rates of otherwise independent physical declines to co-vary between individuals. Such a relationship among multiple physical declines has a precedent in human medicine, being reminiscent of the multi-dimensional clinical presentation of frailty [48] observed in geriatric human populations.

Our hierarchical process model explains the paradoxical “uncoupling” of VMC timing and lifespan by mutations and interventions in aging [17, 23, 24]. Our findings suggest that VMC times and lifespan are not “uncoupled” by mutations and interventions because they are never directly “coupled” to begin with and remain equally “coupled” before and after interventions that disproportionately alter population-mean VMC and lifespan. Our model further suggests that the indirect nature of interactions between VMC and lifespan undermines the use of vigorous movement as a proxy for an organismal “health”. In system well-described by a hierarchical process model, good behavioral health and longevity are experienced not sequentially but in parallel driven by distinct underlying physical declines. In other words, our model suggests that *C*. *elegans* has at least two distinct biological ages, neither of which uniquely reflect a single, organismal “health”.

This study does not attempt to untangle the mechanistic bases of these two aging processes, sufficing only to identify their distinct existences and clarify their causal relationship. Notably, we identify *eat-2(ad1116)* as an exceptional mutation that does eliminate the negative correlation between VMC and remaining lifespan, seeming to restructure the process hierarchy responsible for this correlation. Because food deprivation produces a multifaceted effect on physiology, we do not gain immediate mechanistic insights from *eat-2(ad1116)*. However, we do learn that the structural relationship between the two aging processes determining VMC and lifespan is not immutable. We speculate that food deprivation might act by introducing new limitations to VMC, for example revealing a shared nutritional requirement of vigorous movement and death time invisible in well-fed animals but acting as a direct causal determinant of both VMC and death times when that nutrient becomes limited during food deprivation. The introduction of a shared, direct causal determinant of both VMC and death times would cause the two processes in our model to act effectively as one, eliminating the negative relationship between VMC and remaining lifespan as we observe in *eat-2(ad1116)* populations.

Though we identify two aging processes in *C*. *elegans* by comparing two events—VMC and lifespan—in principle any number of additional processes might be identified by characterizing additional events. Weak movement cessation did not seem to constitute such an event, but nematode aging is phenotypically highly multi-dimentional [7] and it remains of great interest to see how many distinct time-dependent aging processes are at work in nematode aging. Furthermore, if *C*. *elegans* contains at least two distinct aging processes, then mammals with their larger size and greater complexity of self-renewing tissue types seem likely to exhibit an even larger number of coupled aging processes.

**Fig 7:**
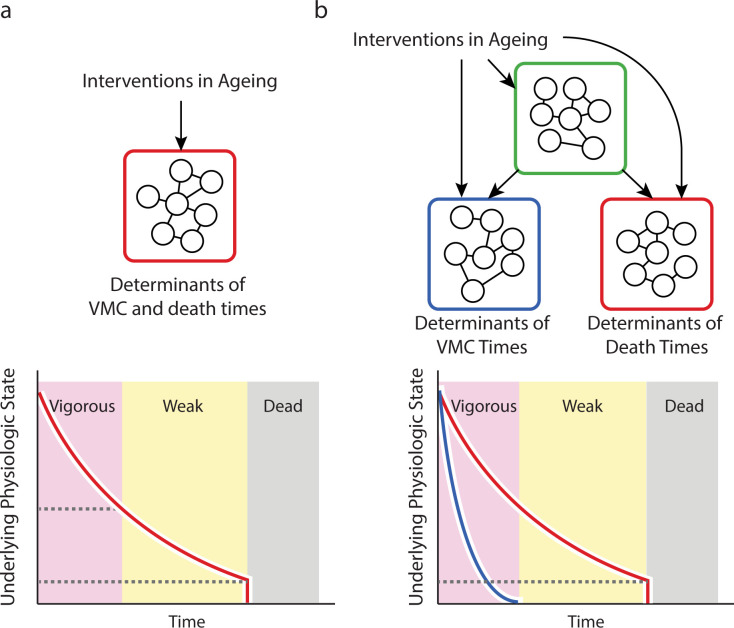
A hierarchical model of vigorous movement and lifespan. a. In a simple, null model, the correlation between vigorous movement cessation (VMC) and death times arises because the two events share physiologic determinants (red box) such that VMC and death represent sequential manifestations of a single underlying aging process (red line in the graph). b. However, our data and modelling suggest that VMC and death times are instead determined by distinct sets of physiologic determinants (red and blue boxes) with each set subject to a distinct aging process (red and blue lines in the graph). In this case, a hierarchical organization among processes allows interventions to act directly on each process, or indirectly through an influence on shared upstream factors (green box).

The potential for complex causal interplay between large numbers of partially independent aging processes calls into question a crucial assumption of aging biomarker development—that a biomarker’s response to intervention will report on same aging underlying biology that mediates its correlation with clinical outcomes in the absence of intervention. Our model demonstrates how, in a multi-process system (Fig 7), biomarkers can predict important outcomes of aging not because they directly report on the process determining those outcomes but because they are indirectly coupled to that processes via shared, systemic factors. When researchers discover a novel intervention that produces compelling effects as measured by a clinically-predictive biomarker, they should not immediately conclude that the novel intervention will affect clinical outcomes. In a multi-process system, the intervention may be acting locally only on the biomarker-determining process, altering its relationship with the systemic factors that previously allowed it to predict outcomes.

Such concerns can be addressed by systematic efforts to map the causal structure of the processes that link biomarkers to outcomes. One approach for this is to employ diverse panels of biomarkers capable of distinguishing multiple, interacting aging processes—ideally this would involve a set of biomarkers highly correlated with outcomes but minimally correlated with each other—just as VMC and death times are partially but not fully correlated. Such a panel could then be analyzed through end-point statistics as done here or, alternately, through longitudinal analysis [49]. If researchers are limited in the biomarkers available for study, they should avoid making implicit assumptions regarding the single-state Markovian behavior of their system and instead focus on testing such assumptions, for example by directly measuring outcomes before and after intervention and explicitly evaluating the contextual limitations of their biomarker. In systems characterized by multiple, partially independent aging processes, many simplifying assumptions used to accelerate research studies will prove dangerous, due to the possibility that even highly-unrelated mechanisms will correlate with outcomes due to coupling with systemic factors.

### Materials and experimental methods

The following nematode strains were used: QZ0 [wild type (Bristol)], QZ120 [*daf-2 (e1368)*], QZ60 [*daf-16(mu86)*], QZ121 [*hsf-1(sy441)*], QZ414 [*eat-2(ad1116)*], and MQ1333 [*nuo-6(qm200)*]. HT1593 [*unc-119(ed3)*], CF1904 [*glp-1 (e2141*)], AMP100 [*rpb*-*2 (cer135); peft-3*::*TIR1*] with *cer135* corresponding the CRISPR-inserted AID tag *rpb-2*::*GFPΔpiRNA*::*AID*::*3xFLAG*.

All animals were housed at 20°C or 25°C at a density of approximately 40 animals per agar plate, seeded with either live OP50 *E*. *coli* or UV-inactivated NEC937 (OP50 ΔuvrA; KanR) [50] *E*. *coli*. Automated imaging was performed on 5 cm plates, and all other vermiculture was performed on 6 cm plates. Figs 1, 2, 3 and 6: 20°C UV-inactivated; Fig 5: UV-inactivated at 20°C except for the 20°C to 25°C which was on UV-inactivated bacteria and the live to UV-inactivated bacteria comparison which was at 25°C. Age synchronous populations were obtained by hypochlorite treatment of gravid populations, except for *eat-2 (ad116)* and *nuo-6 (qm200)* strains in which animals were synchronized by manually selecting late L4 larvae. To eliminate progeny, populations were transferred at late L4 stage onto plates containing 27.5 μg ml^−1^ 5-fluoro-2-deoxyuridine (FUDR, Sigma). Where live bacteria were used, 10 μg ml^−1^ FUDR sufficed to eliminate live progeny. 1-Naphthaleneacetic acid (Sigma-Aldrich), was solubilized in 1M potassium hydroxide and then added into molten agar. *rpb-2* RNAi in S6A Fig was performed using age-synchronous spe-9(hc88) I; rrf-3(b26) animals housed at 25°C in the absence of FUDR, transferring animals at L4 stage from empty vector HT115 onto HT115 bacteria containing the *rpb-2 RNAi* construct. S6B Fig was performed at 20°C on UV-inactivated bacteria. The automated lifespan equipment was calibrated and operated as previously described [28].

By hand annotation of individual worm trajectories used in Fig 1 were performed using the Worm Browser, which is part of the Lifespan Machine software package. All event times, including VMC and death, are counted in reference to the first day of adulthood—twenty for hours after final steps of vulval development of L4 larvae.

### Additional weiner process simulation description

Adopting the assumptions of previous work [9], we considered a set of synthetic individuals that begin “life” identical, and then “decline” according to a one-dimensional discrete time, continuous biased random walk (Weiner process). State transitions—between vigorous movement and death—were defined to occur at the time each individual passes below a certain distance threshold, with subsequent thresholds defining subsequent states (Fig 4A). This type of Weiner process model, in the single-threshold case, has been shown to produce mortality statistics that recapitulate many aspects of biological aging [9].

We modeled a single Wiener process model (Fig 4B) as well as a random walk in which a frailty term [35, 51] was introduced with the effect of giving each individual a distinct drift rate. This frailty mimics the heterogeneity in aging rates observed in populations of *C*. *elegans* and humans, where indistinguishable individuals exhibit consistent, life-long differences in the risk of adverse age-associated events and visible aging phenotypes [2, 7].

To simulate a non-Markovian process, we then considered a Wiener process model with a modified frailty term, such that individuals possess distinct (and inversely correlated) drift terms before and after transitioning from state 1 to state 2 (Fig 4C). Such a model, by construction, produces an inverse correlation between vigorous movement and remaining lifespan.

We then simulated a system of two independent Weiner processes (Fig 4D), in which each stage transition is determined by a distinct process. Finally, we added a frailty effect, similar to Fig 3B), in which the characteristic drift term of each individual is shared between the two processes.

### Additional partial independence simulation description

We modified the two-process frailty model (Fig 4E) to allow a tunable dependence between processes (S4 Fig). As before, at the start of each simulation random walkers start at the same position and then decline following to two biased random walks A and B, with walk A (blue) determining the transition time into state 2 (weak movement), and walk B (red) determining the transition time into state 3 (death). However, at each step of the simulation the two random walkers progress according to the weighted sum of a shared random variable and a process-specific random variable. By altering the weight of this weighted sum, we were able to tune the exact degree of independence between processes A and B.

### Statistical methods

**Transition-Specific Hazard Functions (Figs 1–3)—**The risk of state transitions was calculated as a function of chronological age through numerical differentiation of the Kaplan-Meier cumulative hazard estimate in R. Clock-reset hazard models were calculated by subtracting vigorous movement cessation times from each individual’s lifespan and numerically differentiating the cumulative hazard estimate of that set in R.

**Parametric fits (S1 Fig)—**Parametric fits were estimated using the following forms:

$$\begin{array}{cc} \mathrm{Gompertz}: & h(t|a,b)=\frac{a}{b}\exp(\frac{t}{b})\\ \mathrm{Weibull}: & h(t|\alpha ,\beta )=\frac{\alpha }{\beta }{\left(\frac{t}{\beta }\right)}^{\alpha -1}\\ \mathrm{Weibull}\;\mathrm{with}\;\mathrm{frailty:} & h(t|\alpha ,\beta ,\sigma )=\frac{\frac{\alpha }{\beta }{\left(\frac{t}{\beta }\right)}^{\alpha -1}}{1+\sigma^{2}{\left(\frac{t}{\beta }\right)}^{\alpha }}\\ \mathrm{Inverse}\;\mathrm{Gaussian:} & h(t|\mu ,\lambda )=\frac{\sqrt{\frac{\lambda }{2\pi t^{3}}}\exp(-\frac{\lambda {\left(t-\mu \right)}^{2}}{2\mu^{2}t})}{1-\Phi (\sqrt{\frac{\lambda }{t}}(\frac{t}{\mu }-1))-\exp(\frac{2\lambda }{\mu })\phi (-\sqrt{\frac{\lambda }{t}}(\frac{t}{\mu }+1))} \end{array}$$

A more in-depth description of these parameterizations is available in [33]. All parametric fits, along with parametric regression models, were performed using the flexsurv [52] package in R.

**Comparison between state transition times (Fig 1–3)—**The relationship between state transition times was explored using the multiple regression model with which we control for the confounding influence of batch effects:

$$d_{i}=\beta_{V}v_{i}+\beta_{R}R_{i}+\beta_{\mathrm{cross}}X_{i}R_{i}+c$$

where *v*_*i*_ is the time of cessation of vigorous movement and *d*_*i*_ is the death time of individual *i*. *R*_*i*_ is a categorical variable coding for the batch of individual *i* which in all cases represented the specific flatbed scanner on which the individual was observed. A variety of residual forms and link functions were explored using a generalized linear model approach. We found that standard linear regression provided the best performance, and so the regression was solved using rlm using the ‘RMS’ [53] package in R. Partial coefficients of determination were calculated by comparing the full model with a reduced model with the reduced model *y_i_ = β_R_R_i_ + α* using the r package ‘rsq’ [54]. A more in depth exploration of this regression approach is presented in the supporting texts S1 Text and S2 Text. The same model was run to evaluate weak movement span, with *v*_*i*_ then representing the timing of cessation of weak movement. LOESS regression was performed in R.

**Stochastic process simulations (Fig 4)—**We simulated a one dimensional Weiner processes with drift that satisfies the property that between times *t* and *t + Δt*, individuals advance in their positions *W(t)* such that

$$W(t+\Delta t)-W(t)=N(\mu \Delta t,\sigma^{2}\Delta t)$$

where N is the normal distribution representing a drift of μ relative to the variance σ^2^. Each simulation considered the progression of 1000 random walkers, starting at a displacement from the origin of about 4x10^5^ times larger than the drift term. Time was segmented into even steps, and in each step each walker advanced towards zero according to the above *W*(*t*+Δ*t*)−*W*(*t*) property. The state transition time of each individual was identified as the first time step in which a walker’s position fell below a constant threshold. For single process models, sequential state transitions correspond to walkers’ passing sequentially lower thresholds. For process models involving frailty, each random walker was assigned a characteristic drift rate, distributed according to a gamma distribution with equal shape and scale parameters, centered around the population average drift-rate μ. For multiple process models, walkers progressed according two independent Weiner processes, each with its own drift $\mu$ and threshold governing state transition.

**Stochastic process simulations (S4 Fig)—**The same simulation structure was used as in main text Fig 4, but this time the step sizes are defined in respect to an additional parameter *w*, such that

$$\begin{array}{c} W_{A}(t+\Delta t)-W_{A}(t)=(1-w)N(\mu_{X}\Delta t,(1-w)\sigma^{2}\Delta t)+wN(0,w\sigma^{2}\Delta t)\\ W_{B}(t+\Delta t)-W_{B}(t)=(1-w)N(\mu_{Z}\Delta t,(1-w)\sigma^{2}\Delta t)+wN(0,w\sigma^{2}\Delta t) \end{array}$$

where each N(μ,σ) is an independent Gaussian random variable. As can be seen, the means of these variables do not depend on *w*, but their variances do depend on *w*. This configuration yields a model for which the mean and variance of $W_{A}(t+\Delta t)-W_{A}(t)$ and $W_{B}(t+\Delta t)-W_{B}(t)$ are independent of *w*, which can be shown using the property of Gaussian random variables that N(m,n)+N(o,p)=N(m+o,n+p). State transition times are identified as the first step in which *W*_*A*_(*t*) and *W*_*B*_(*t*) fall below their respective thresholds.

**Accelerated Failure Time models (Figs 5 and 6)—**Scale factors were estimated for each intervention, estimating its effect on vigorous movement cessation time, weak movement cessation time, and death times separately. In each case the following model was fit:

$$\log(y_{i})=\beta_{X}X_{i}+\beta_{R}R_{i}$$

where *y*_*i*_ is the event time under consideration, *X*_*i*_ is a categorical dummy variable coding for the genotype or environmental condition being evaluated, and *R*_*i*_ is the batch effect (in this case the flatbed scanner on which the individual was housed).

## Supporting information

S1 Fig Automated, high-throughput phenotyping. **a.** Using hand-annotated trajectories for 830 wild-type animals whose lifespan exposed to variety of environmental conditions, we built a Hidden Markov Chain Model that estimates probability of all possible transitions among states. Shown are the state transition probabilities for an individual after six hours spent in the current state. **b.** The results of a six-fold cross-validation scheme—the same data as in Fig 1*D* and 1*E* but plotted as Kaplan-Meier survival curves separately for each independent biological replicate, with by-hand (black) and automated results (red) compared. **c.** The error for each death time in these survival curves, plotted as a cumulative distribution function for each replicate. **d.** For the population of wild-type animals considered in Fig 1*B* and 1*C*, the cumulative distribution function describing the time spent non-moving prior to death and **e.** the fraction of lifespan spent non-moving.
(PDF)

S2 Fig Relating vigorous movement and lifespan in wild-type populations. **a.** The "clock-reset" hazard function showing the risk of death as a function of time after weak movement cessation (WMC). **b.** The absolute value of the residuals from the regression model d_i_ = v_i_+ ε_i_, with the LOESS regression line (red) and 95 confidence bands for that interval (pink) and the mean residual value (blue). The White test statistic had a value of 28, showing a significant deviation from homoscedasticity at p = .015. **c.** the absolute value of the residuals from the regression model d_i_ = v_i_+ ε_i_, with the LOESS and mean residual data shown as before. The White test statistic has a value of 233, larger than before, showing a deviation from homoscedasticity at p < 10^^-10^. **d.** The relationship between VMC and death times for each of 10 biological replicates of wild-type lifespan experiments, with linear regression lines (red) and LOESS regression line (green) overlaid. **e.** The same analysis, but comparing VMC to the lifespan remaining after VMC.**f.** The slope of the linear regression line, β_v_, relating VMC to lifespan as shown in panel d, compared across all replicates, grouped by food source (left) or environmental temperature (right).**g.** The same analysis as main text Fig 2C, comparing VMC and death times, in a single population of wild-type animals at 20°C, but here excluding individuals with the top and bottom 5th percentiles (gray dotted lines) of death times (left), of VMC times(middle) or of both VMC and death times (right).
(PDF)

S3 Fig The effect of interventions on vigorous movement and lifespan. **a-b**. We replicate the analysis of main text Fig 3I and 3J comparing the vigorous movement cessation (VMC) death times, but this time for a population exposed to a lower concentration, 1.5 mM t-BuOOH. **c.** For all interventions considered in main text Fig 5, we present the linear regression estimates relating VMC and death times. Significant deviations from β_v_ = 1 were estimated by bootstrapping and marked with a star. **d.** A comparison between VMC and death times for the two *eat-2(ad1116)* replicates as well as the bacterial deprivation experiment, along with the corresponding wild-type controls, with linear regression (red) *x = y* unity lines (gray) overlaid. **e.** The same analysis and conditions, but this time comparing VMC times to remaining lifespan after VMC, with linear regression lines (red) and LOESS regression curves (green).
(PDF)

S4 Fig Stochastic models of aging link VMC and death times. To test our ability to infer dependencies among biological processes using only state transition times, we developed a model that allows us to vary the interdependence between two biased random walks (Weiner processes). **a.** In this model, the step size of two Weiner process *A* (red) and *B* (blue) at each step of the simulation are determined as the weighted sum of random variables *X* and *Y*, and the weighted sum of *Y*, and Z respectively. As in main text Fig 4E, the drift term of both processes vary between individuals to simulate frailty. By changing the weight *w*, the relative contribution of the independent components *X*, *Z* and the shared component *Y* can be adjusted. **b.** When *w* is set to 1, the two Weiner processes are determined entirely by the shared upstream component *Y* and the model yields both a positive correlation between state exit times (left) and a positive correlation between state 1 exit time and the duration of state 2(right). **c.** The same simulation results plotted, but with *w* set to 0.8. **d.** The same relationships produced when *w* is set to 0.6, **e.** with *w* set to 0.4, **f.** with *w* set to 0.2, and **g.** with *w* set to 0. At *w* = 0, *A* and *B* are completely independent and the simulation is equivalent to main text Fig 4E. **h.** Across a range of *w* values, we compare the relationship between state 1 exit ties and state 2 duration, β_v_, roughly corresponding to the relationship between VMC time and remaining lifespan observed in our experimental data.
(PDF)

S5 Fig The relative effects of mutations and interventions on vigorous movement and lifespan. **a.** The proportional and disproportionate effects of each intervention and mutations shown in Fig 5 were estimated (S5 Text), with Δμ_s_ quantifying the proportional action and R_f_ quantifying the disproportionate action of each intervention on VMC and death times.
(PDF)

S6 Fig An RNA Polymerase II dosage series. **a.** On the second day of adulthood, animals living on HT115 at 25°C were transferred from empty vector to *rpb-2* RNAi and the Kaplan-Meier survival curve was estimated. **b.** *rpb-2(+);peft-3*::TIR1 populations were exposed to either 0 and 8 mM α-Naphthaleneacetic acid (NA) starting on day 2 of adulthood. Lifespan is shown as Kaplan-Meier survival estimates **c.** In a separate biological replicate, the effects of NA on *rpb-2*(+); *peft-3*::TIR1 (circles) and *rpb-2*::AID; *peft-3*::TIR1 (squares) were compared, via the AFT-regression parameters estimated for VMC and lifespan remaining after VMC.. 6d. Error bars indicate 95% confidence intervals. **d.** At each NA concentration, R_f_ and Δμ_s_ were calculated to quantify the magnitude of disproportionate and proportionate changes, respectively, of NA on VMC and lifespan (S5 Text), for *rpb-2(+);peft-3*::*TIR1* (circles) and *rpb-2*::AID;*peft-3*::TIR1 (squares) populations. Error bars indicate 95% confidence intervals.
(PDF)

S1 Text Lifespan Machine Technology Update. A description of the new approaches to image analysis, including detection of death-associated contraction and expansion and partitioning of lifespan into distinct behavioral and morphological stages.
(PDF)

S2 Text Age-associated Risk Trajectories of VMC and death. A parametric approach to quantifying similarities and differences in the time-dependent dynamics of the risk of vigorous movement cessation and death.
(PDF)

S3 Text Process models of VMC and death times. Formal specification of the process model relating vigorous movement cessation and lifespan, and its relationship to the slope of the linear regression line relating vigorous movement cessation and death times.
(PDF)

S4 Text Accounting for environmental effects and measurement error. An exploration of alternate hypothesis for why the slope of the linear regression line relating vigorous movement cessation and death times would be less than one.
(PDF)

S5 Text Quantifying proportional and disproportional effects of interventions on lifespan and VMC. A description of the regression model used to characterize the differential effects of interventions on vigorous movement cessation and lifespan.
(PDF)
