# Supplementary figures and images for: A hierarchical process model links behavioral aging and lifespan in C. elegans
Source: PLoS Comput Biol. 2022 Sep 30;18(9):e1010415. doi: 10.1371/journal.pcbi.1010415 (PMC9524676; doi:10.1371/journal.pcbi.1010415)

a

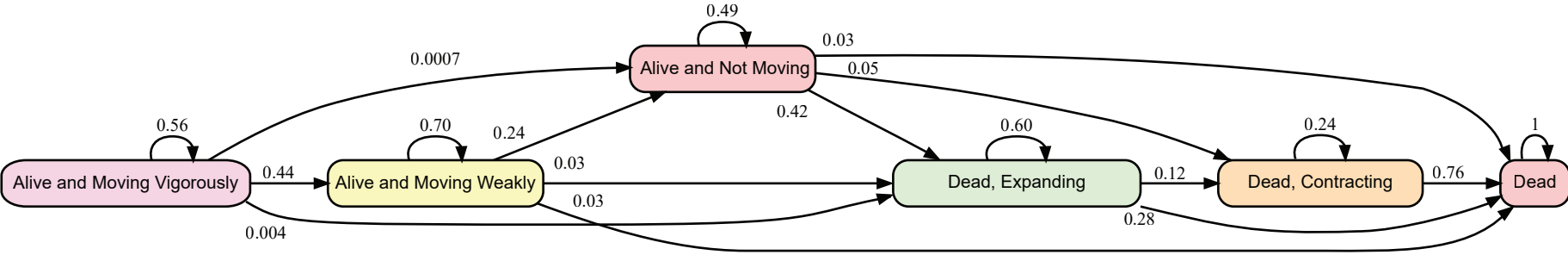

b

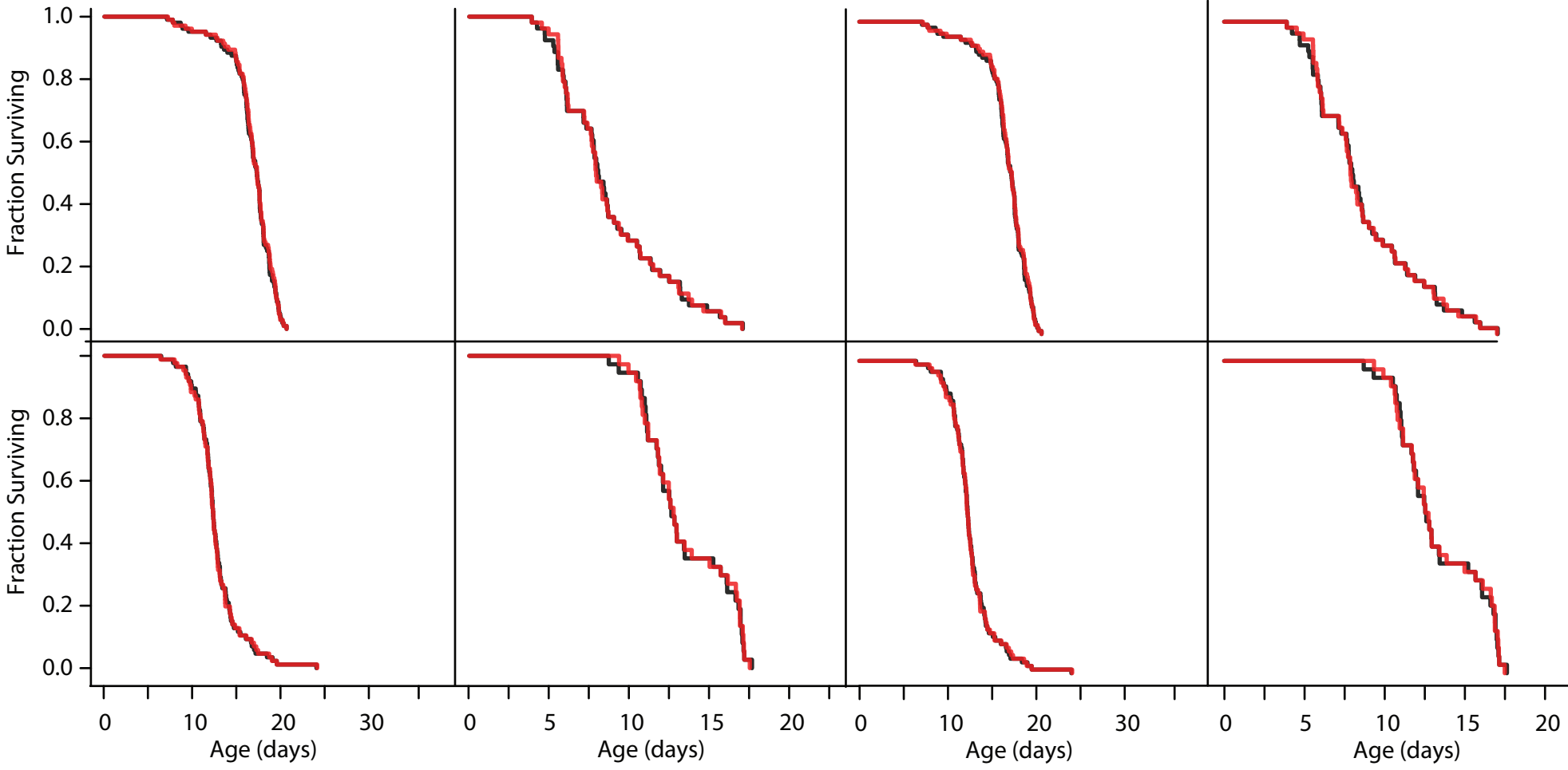

c

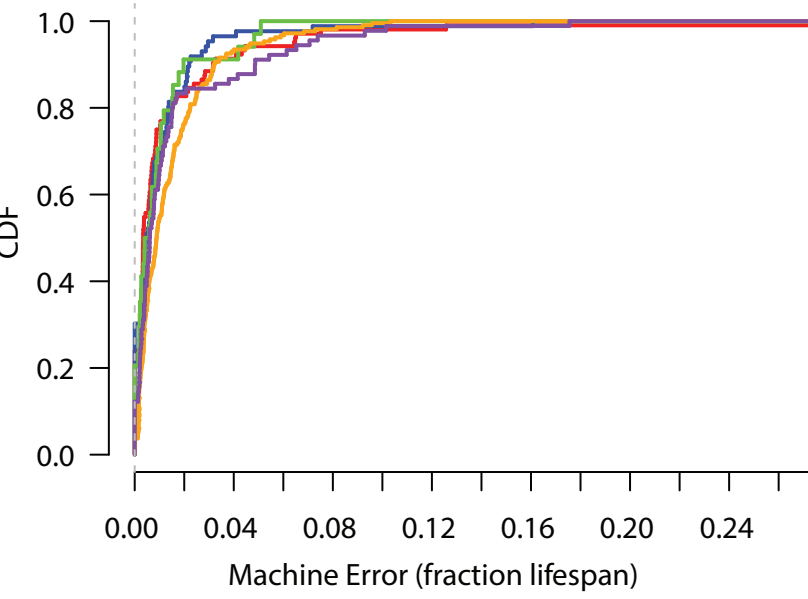

d

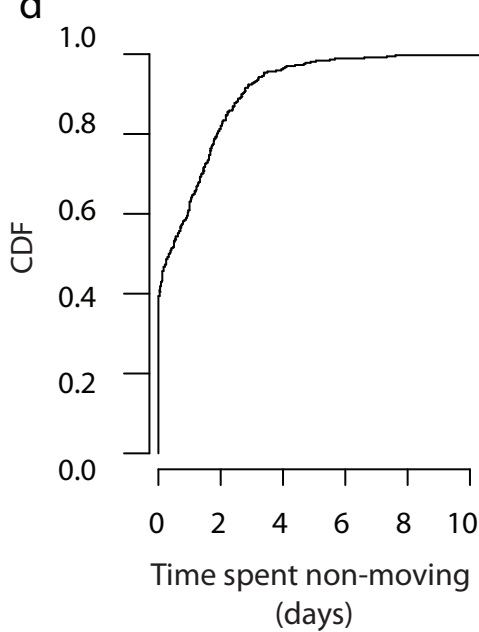

e

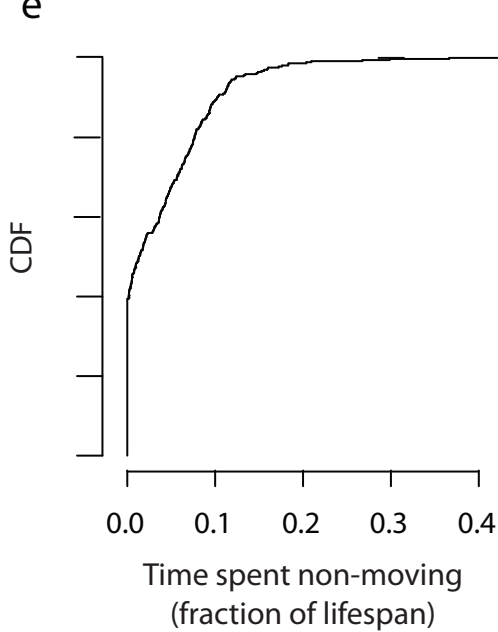

Supplement: S1 Fig — a. Using hand-annotated trajectories for 830 wild-type animals whose lifespan exposed to variety of environmental conditions, we built a Hidden Markov Chain Model that estimates probability of all possible transitions among states. Shown are the state transition probabilities for an individual after six hours spent in the current state. b. The results of a six-fold cross-validation scheme—the same data as in Fig 1D and 1E but plotted as Kaplan-Meier survival curves separately for each independent biological replicate, with by-hand (black) and automated results (red) compared. c. The error for each death time in these survival curves, plotted as a cumulative distribution function for each replicate. d. For the population of wild-type animals considered in Fig 1B and 1C, the cumulative distribution function describing the time spent non-moving prior to death and e. the fraction of lifespan spent non-moving. (PDF) [file pcbi.1010415.s001.pdf]

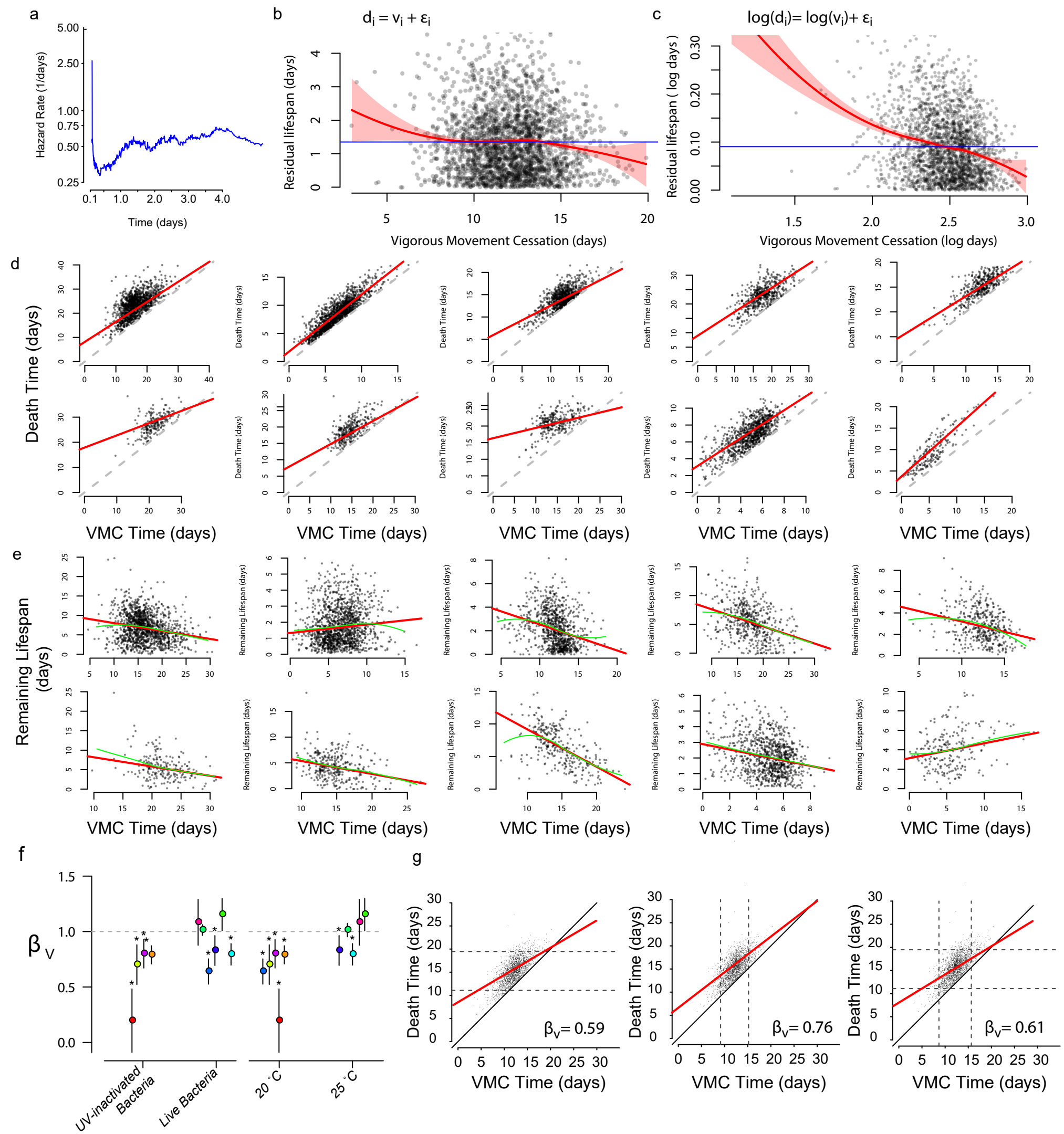

Supplement: S2 Fig — a. The "clock-reset" hazard function showing the risk of death as a function of time after weak movement cessation (WMC). b. The absolute value of the residuals from the regression model di = vi+ εi, with the LOESS regression line (red) and 95 confidence bands for that interval (pink) and the mean residual value (blue). The White test statistic had a value of 28, showing a significant deviation from homoscedasticity at p = .015. c. the absolute value of the residuals from the regression model di = vi+ εi, with the LOESS and mean residual data shown as before. The White test statistic has a value of 233, larger than before, showing a deviation from homoscedasticity at p < 10^-10. d. The relationship between VMC and death times for each of 10 biological replicates of wild-type lifespan experiments, with linear regression lines (red) and LOESS regression line (green) overlaid. e. The same analysis, but comparing VMC to the lifespan remaining after VMC.f. The slope of the linear regression line, βv, relating VMC to lifespan as shown in panel d, compared across all replicates, grouped by food source (left) or environmental temperature (right).g. The same analysis as main text Fig 2C, comparing VMC and death times, in a single population of wild-type animals at 20°C, but here excluding individuals with the top and bottom 5th percentiles (gray dotted lines) of death times (left), of VMC times(middle) or of both VMC and death times (right). (PDF) [file pcbi.1010415.s002.pdf]

a

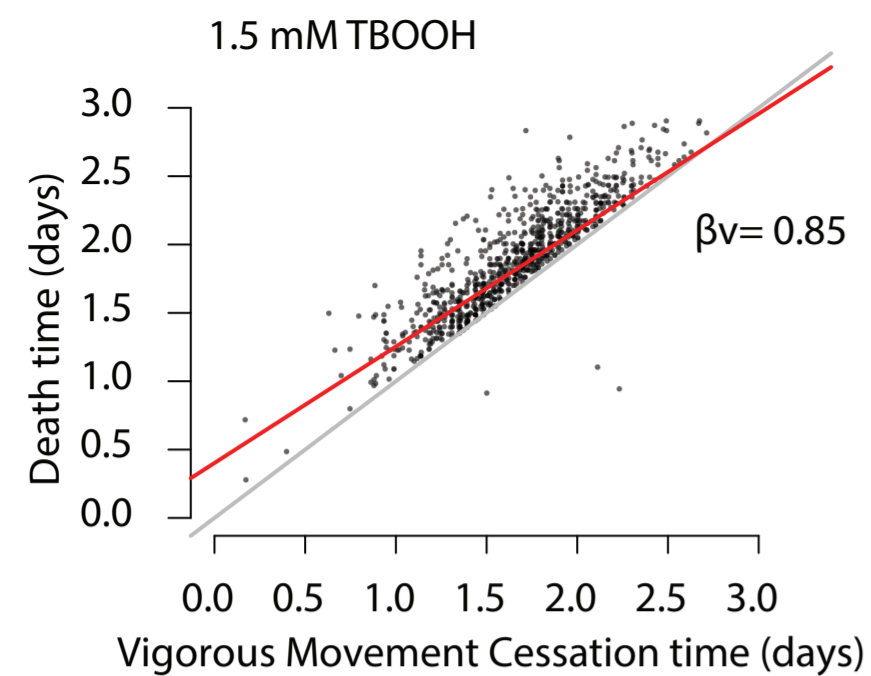

b

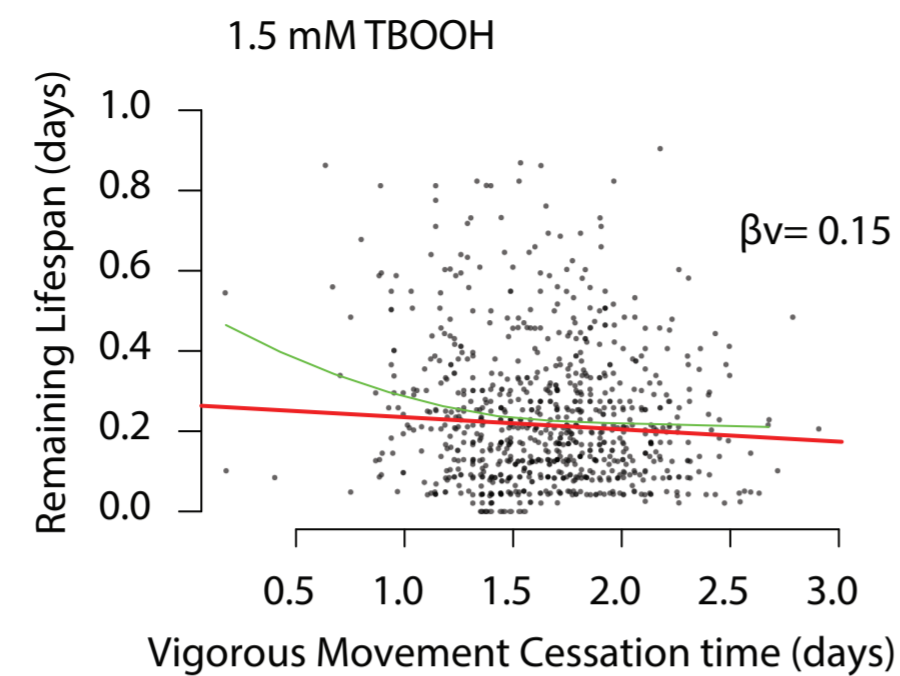

c

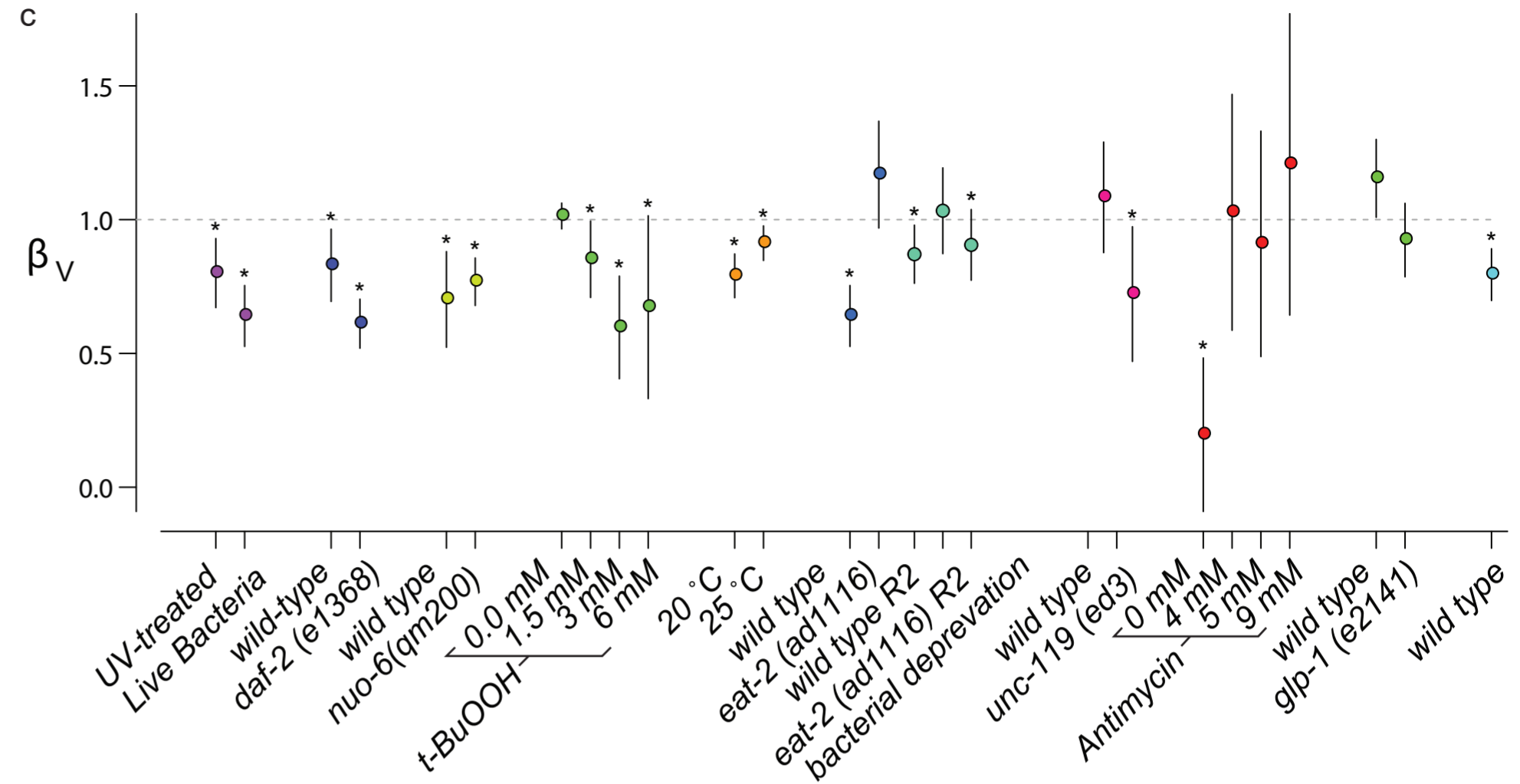

d

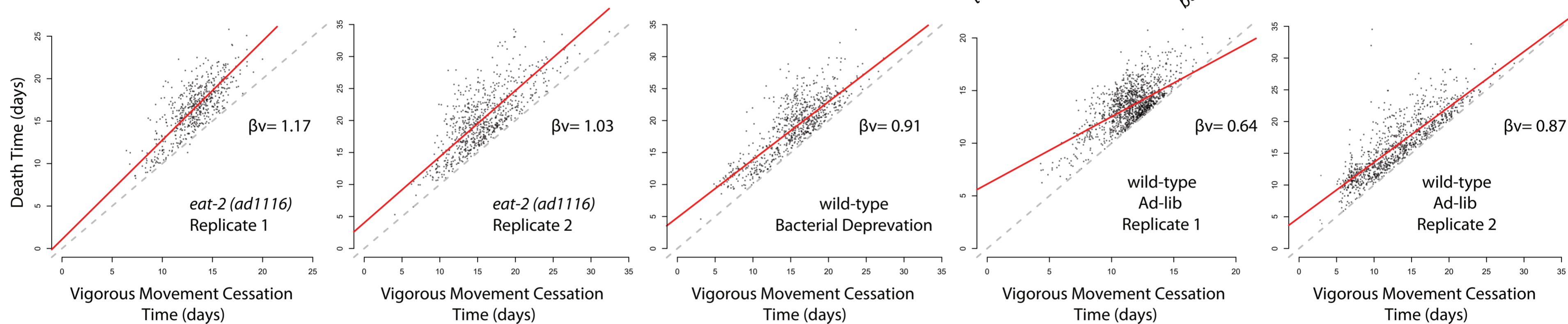

e

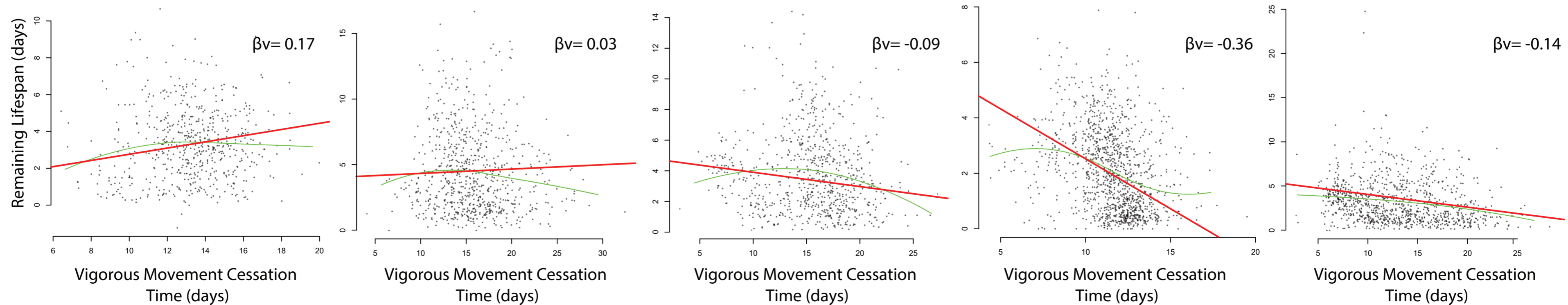

Supplement: S3 Fig — a-b. We replicate the analysis of main text Fig 3I and 3J comparing the vigorous movement cessation (VMC) death times, but this time for a population exposed to a lower concentration, 1.5 mM t-BuOOH. c. For all interventions considered in main text Fig 5, we present the linear regression estimates relating VMC and death times. Significant deviations from βv = 1 were estimated by bootstrapping and marked with a star. d. A comparison between VMC and death times for the two eat-2(ad1116) replicates as well as the bacterial deprivation experiment, along with the corresponding wild-type controls, with linear regression (red) x = y unity lines (gray) overlaid. e. The same analysis and conditions, but this time comparing VMC times to remaining lifespan after VMC, with linear regression lines (red) and LOESS regression curves (green). (PDF) [file pcbi.1010415.s003.pdf]

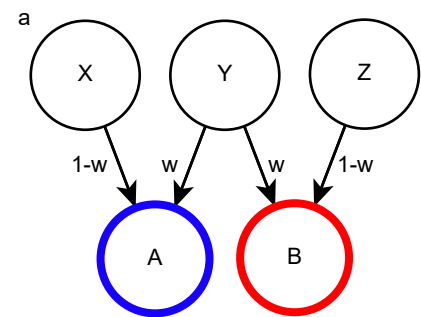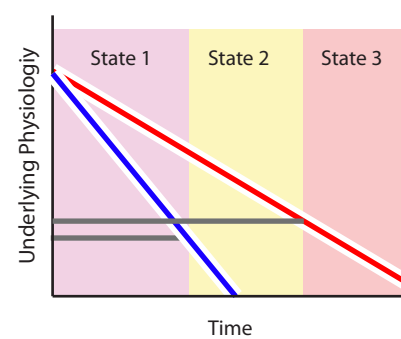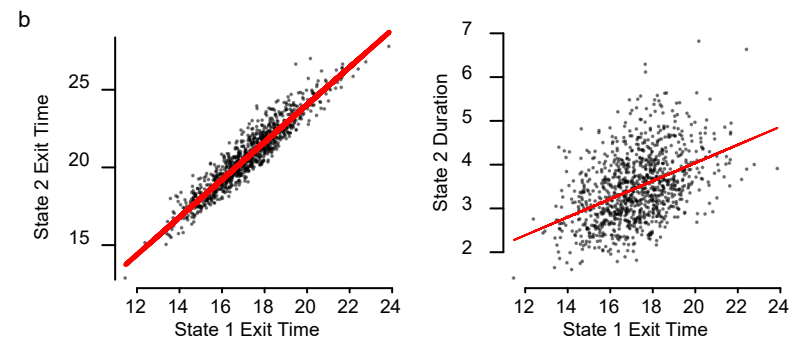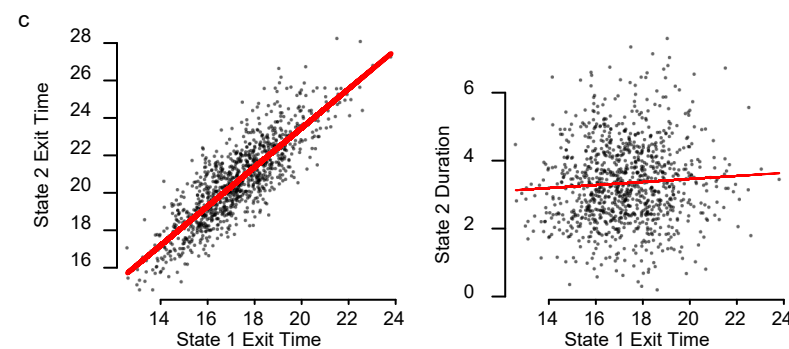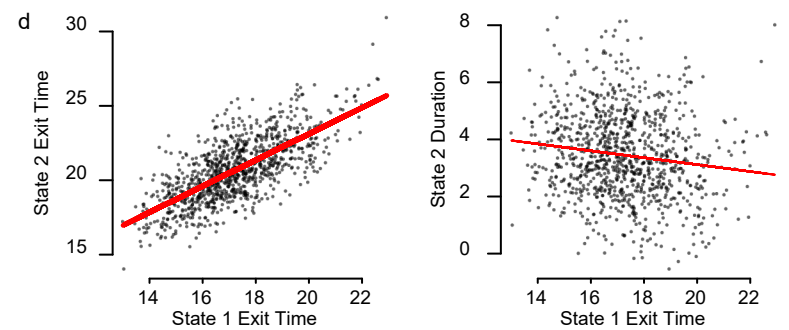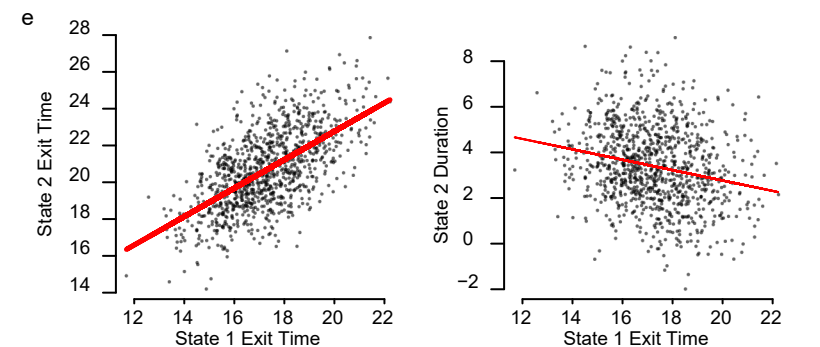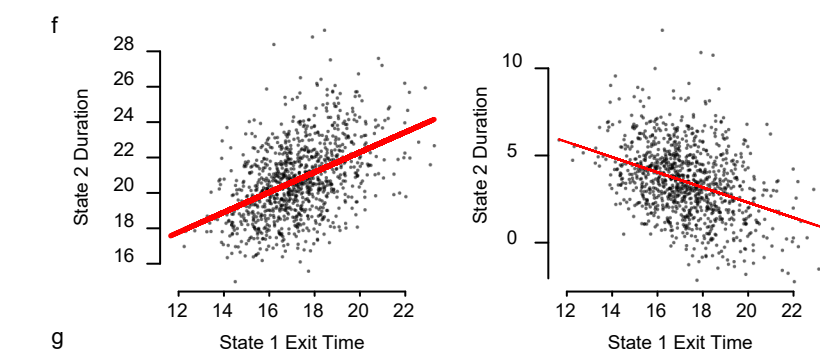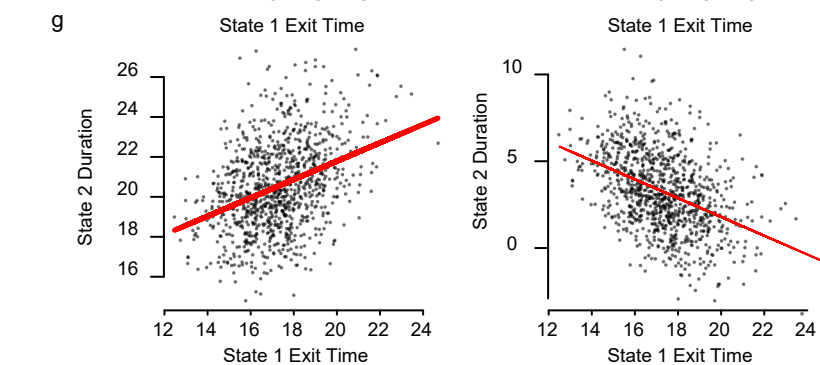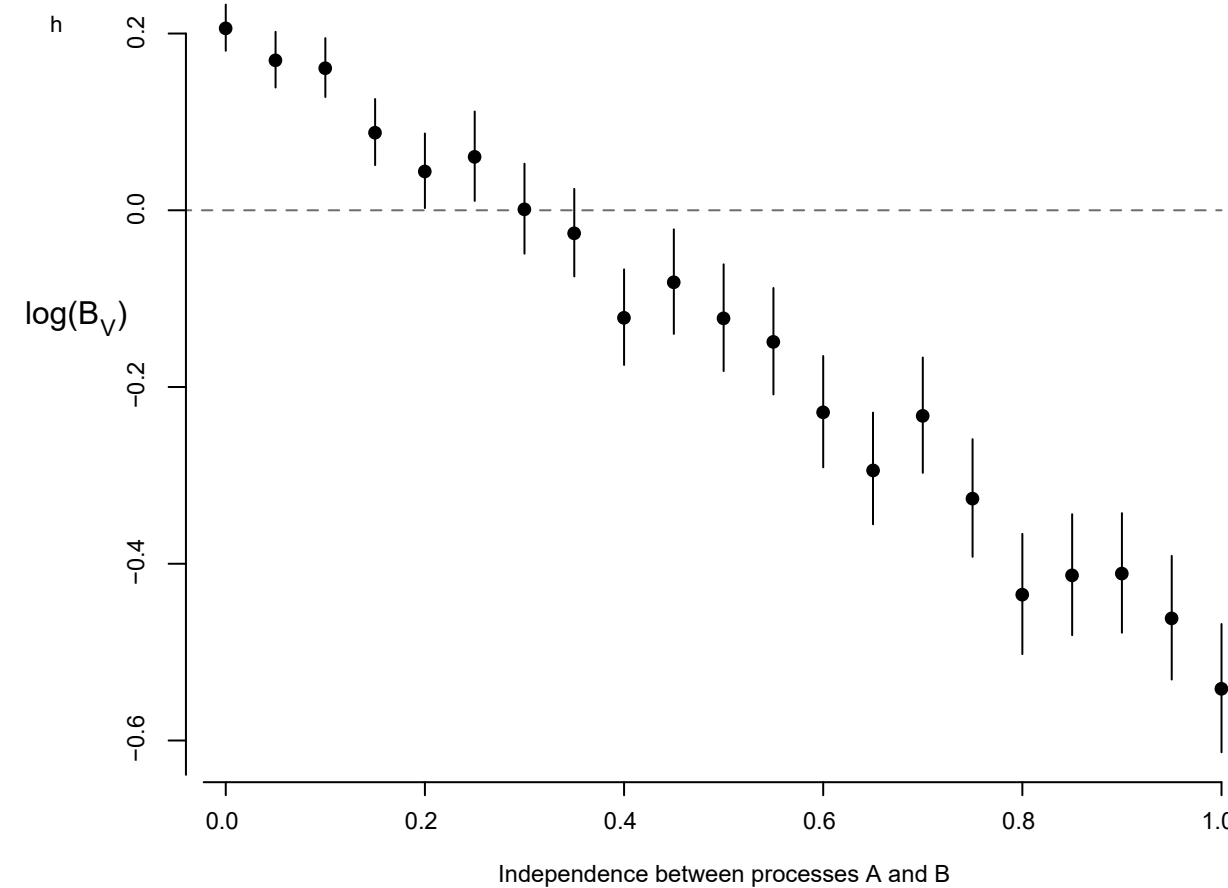

Supplement: S4 Fig — To test our ability to infer dependencies among biological processes using only state transition times, we developed a model that allows us to vary the interdependence between two biased random walks (Weiner processes). a. In this model, the step size of two Weiner process A (red) and B (blue) at each step of the simulation are determined as the weighted sum of random variables X and Y, and the weighted sum of Y, and Z respectively. As in main text Fig 4E, the drift term of both processes vary between individuals to simulate frailty. By changing the weight w, the relative contribution of the independent components X, Z and the shared component Y can be adjusted. b. When w is set to 1, the two Weiner processes are determined entirely by the shared upstream component Y and the model yields both a positive correlation between state exit times (left) and a positive correlation between state 1 exit time and the duration of state 2(right). c. The same simulation results plotted, but with w set to 0.8. d. The same relationships produced when w is set to 0.6, e. with w set to 0.4, f. with w set to 0.2, and g. with w set to 0. At w = 0, A and B are completely independent and the simulation is equivalent to main text Fig 4E. h. Across a range of w values, we compare the relationship between state 1 exit ties and state 2 duration, βv, roughly corresponding to the relationship between VMC time and remaining lifespan observed in our experimental data. (PDF) [file pcbi.1010415.s004.pdf]

a.

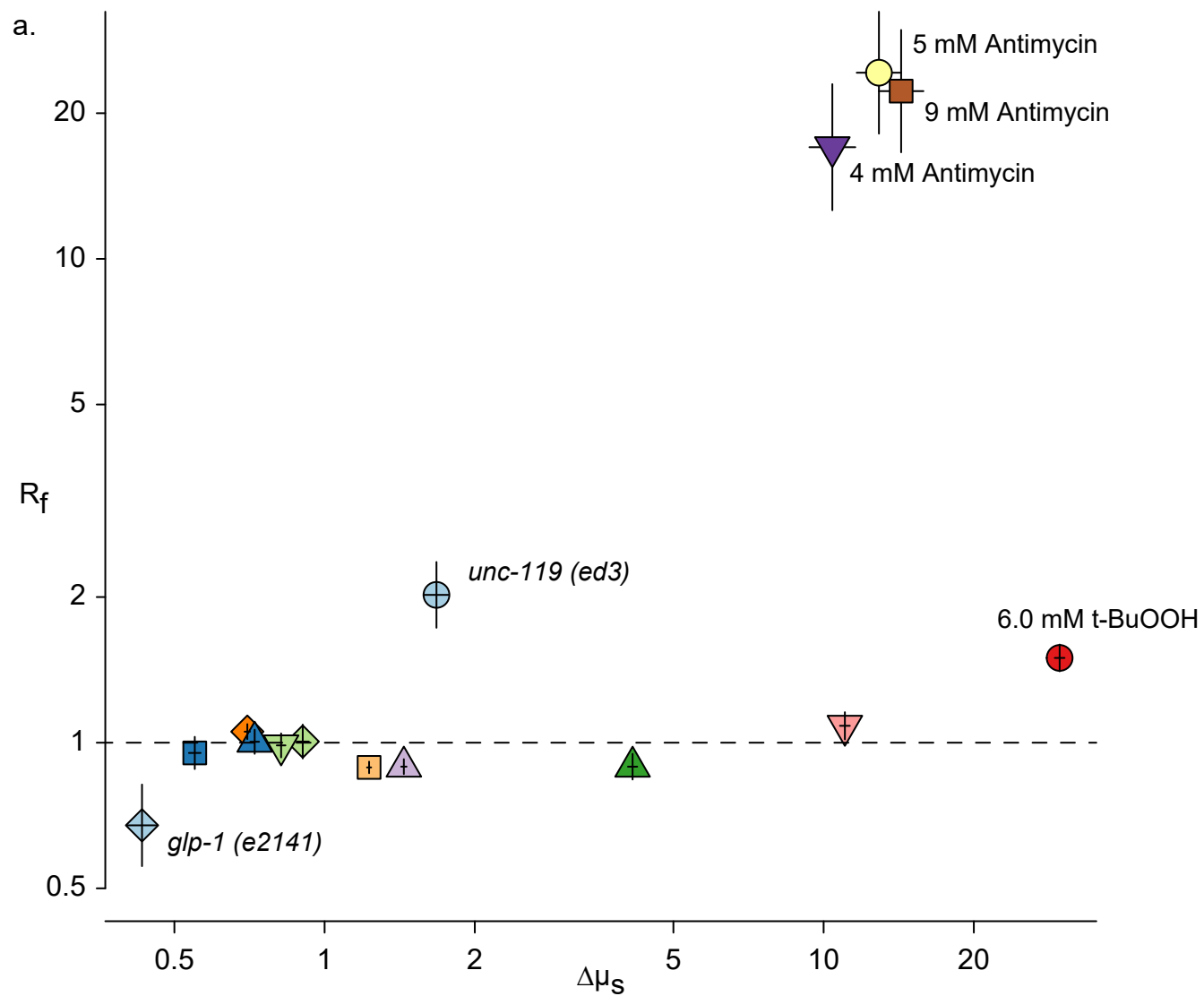

Supplement: S5 Fig — a. The proportional and disproportionate effects of each intervention and mutations shown in Fig 5 were estimated (S5 Text), with Δμs quantifying the proportional action and Rf quantifying the disproportionate action of each intervention on VMC and death times. (PDF) [file pcbi.1010415.s005.pdf]

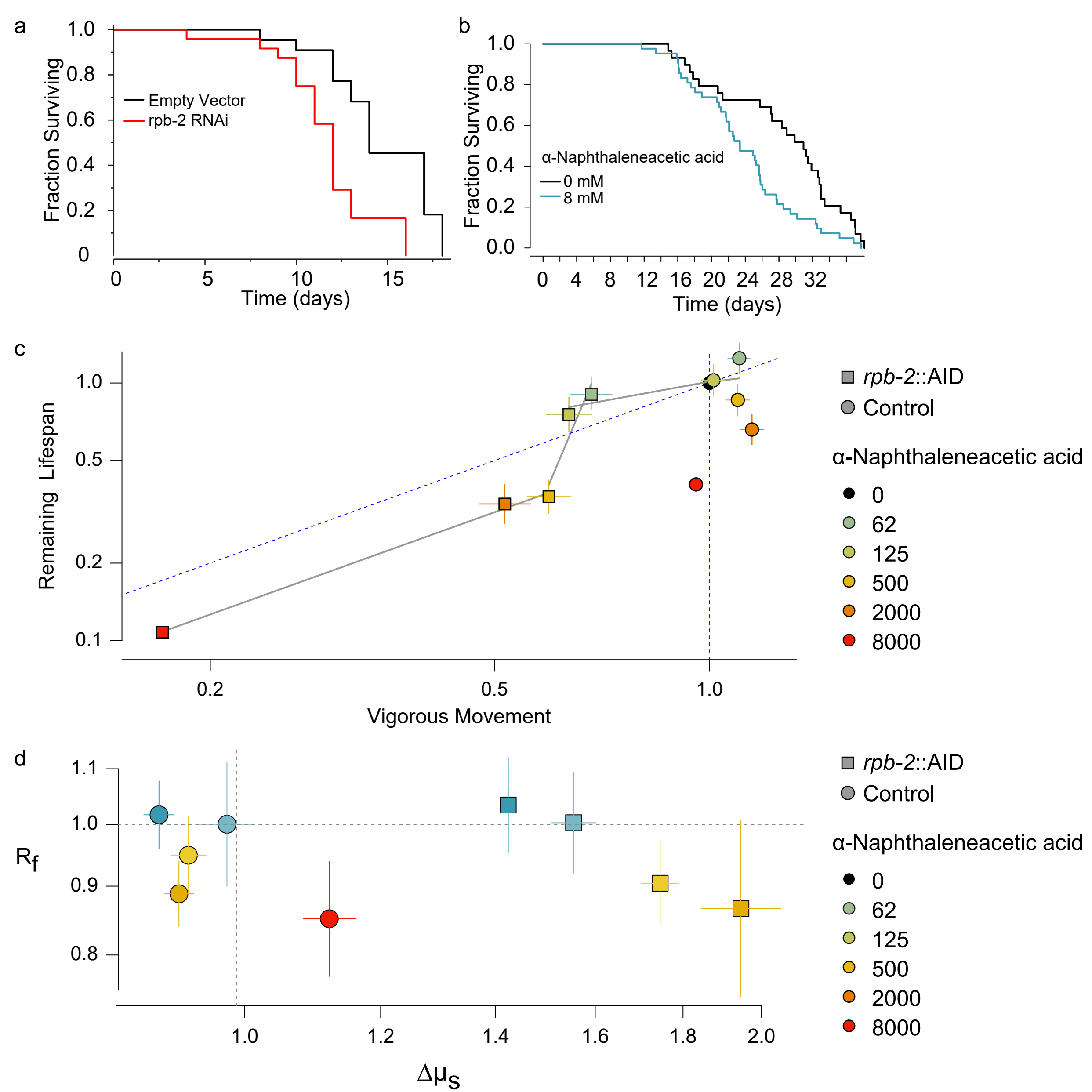

Supplement: S6 Fig — a. On the second day of adulthood, animals living on HT115 at 25°C were transferred from empty vector to rpb-2 RNAi and the Kaplan-Meier survival curve was estimated. b. rpb-2(+);peft-3::TIR1 populations were exposed to either 0 and 8 mM α-Naphthaleneacetic acid (NA) starting on day 2 of adulthood. Lifespan is shown as Kaplan-Meier survival estimates c. In a separate biological replicate, the effects of NA on rpb-2(+); peft-3::TIR1 (circles) and rpb-2::AID; peft-3::TIR1 (squares) were compared, via the AFT-regression parameters estimated for VMC and lifespan remaining after VMC.. 6d. Error bars indicate 95% confidence intervals. d. At each NA concentration, Rf and Δμs were calculated to quantify the magnitude of disproportionate and proportionate changes, respectively, of NA on VMC and lifespan (S5 Text), for rpb-2(+);peft-3::TIR1 (circles) and rpb-2::AID;peft-3::TIR1 (squares) populations. Error bars indicate 95% confidence intervals. (PDF) [file pcbi.1010415.s006.pdf]
