# Supplementary material for: A hierarchical process model links behavioral aging and lifespan in C. elegans
Source: PLoS Comput Biol. 2022 Sep 30;18(9):e1010415. doi: 10.1371/journal.pcbi.1010415 (PMC9524676; doi:10.1371/journal.pcbi.1010415)
Supplement: S1 Text — A description of the new approaches to image analysis, including detection of death-associated contraction and expansion and partitioning of lifespan into distinct behavioral and morphological stages. (PDF) [file pcbi.1010415.s007.pdf]

1 Supporting Text 1–Lifespan Machine Technology Update

1.1 Death-Associated Contraction and Expansion are robust markers of death

At the start of technology development, we had two main goals: first to improve the performance of the lifespan machine and second differentiate more explicitly between death and late-life paralysis which may masquerade as death. We hypothesized that we could reach both goals by developing the death-associated contraction and expansion observed contemporaneously with death[1] as an event for automatic identification.

Therefore, we asked whether death-associated contraction and expansion represent a general feature of nematode death, useful as a proxy for death in a broad set of contexts. We used the lifespan machine to observe the lives and deaths of populations of *C. briggsae*, *C. tropicalis*, *C. japonica*, *C. brenneri* and *P. pacificus* nematodes, whose divergent developmental trajectories[2] reflect a common ancestor living at least 100 million years ago[3], and repeated the functional data analysis approach to characterize death-associated contraction and expansion. We found that, on average, *C. elegans* and *C. briggsae* show nearly indistinguishable trajectories of death-associated contraction and expansion (Panel a in Fig A in S1 Text). To identify quantitative differences between the two species, we considered the eigenvalues associated with the first PCA harmonic, which describes the shared magnitude of contraction and expansion. Despite inter-individual differences within each species, the overall distribution of magnitudes was indistinguishable between *C. elegans* and *C. briggsae* (Panel b in Fig A in S1 Text). We then considered the predatory nematode *P. pacificus*, which also exhibited a similar shape and magnitude of death-associated expansion and contraction (Panels c-d in Fig A in S1 Text). We additionally found that *C. tropicalis* (Panels e-f in Fig A in S1 Text), *C. brenneri* strains (Panels g-h in Fig A in S1 Text), and *C. japonica* (Panels i-j in Fig A in S1 Text), exhibited qualitatively similar death-associated contraction times, whose typical magnitude (Panels e-h in Fig A in S1 Text) varied in relation to *C. elegans*. From these results, we conclude that death-associated contraction and expansion are a general feature of death that was likely present in the earliest common ancestor of these species.

To understand the influence of neuromuscular-driven movement on death-associated contraction, we next considered the effect of two paralytic interventions: an *unc-119 (ed3)* mutation that interferes with neuronal development and causes severe paralysis and Antimycin A which inhibits oxidative phosphorylation and produces severe paralysis. Considering a previously published data set1, we found that *unc-119 (ed3)* animals showed only a slight decrease in the magnitude of death-associated contraction and expansion (Panels k-l in Fig A in S1 Text). Treatment with a 9 mM Antimycin A, an inhibitor of oxidative phosphorylation and a strong paralytic, produced an unexpected increase in the magnitude of death-associated expansion (Panels m-n in Fig A in S1 Text). We conclude that different methods for achieving paralysis can produce different quantitative effects on death-associated contraction, but that even strong paralytic effects do not abolish the phenomenon entirely.

Our data show that death-associated contraction and expansion can provide a, non-behavioral, visible proxy for identifying nematode death. Death-associated contraction is particularly useful for automated methods because, as a non-behavioral event, it does not require external stimulation such as vibration or exposure to blue light[4] to induce movement.

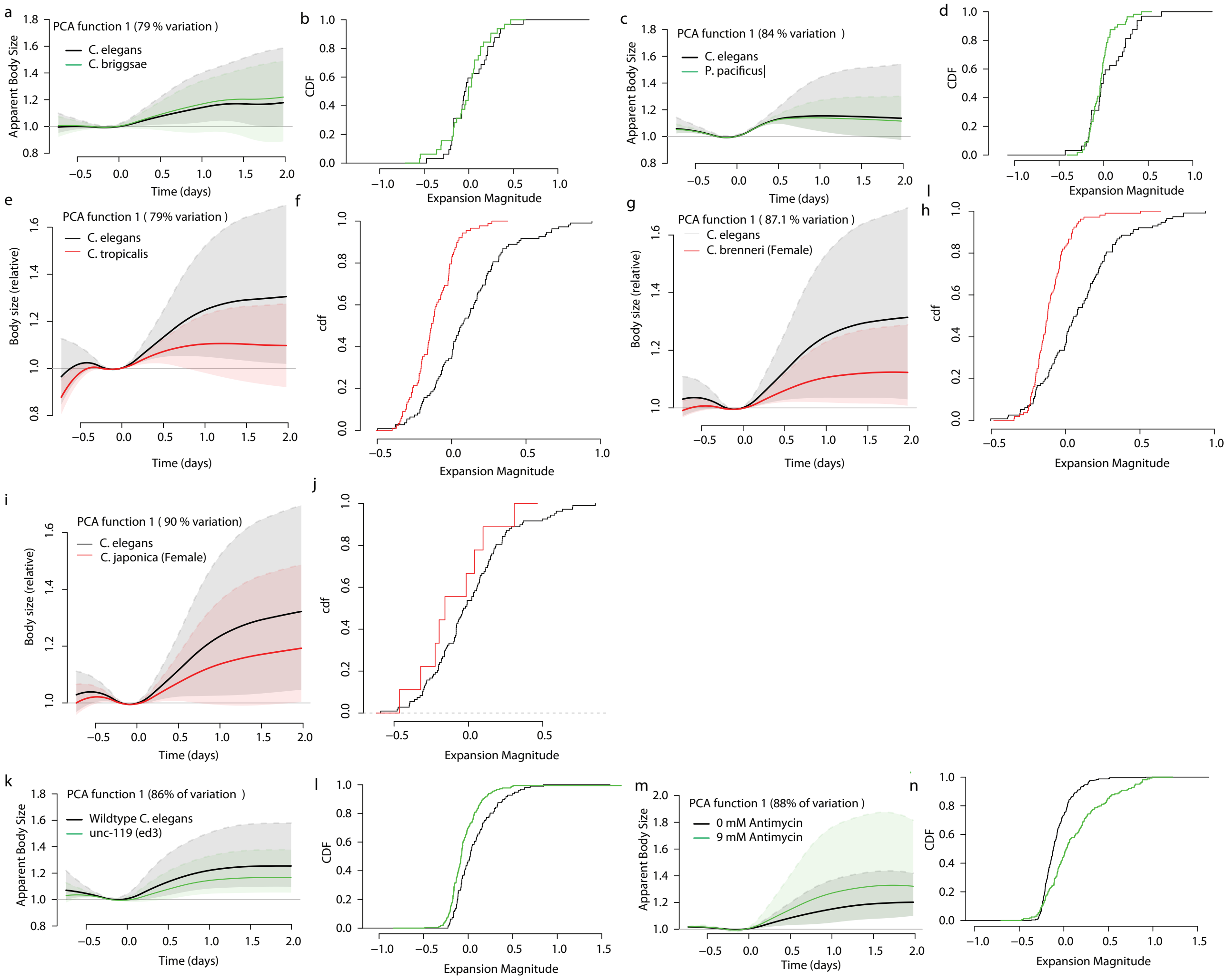

Fig A in S1 Text: Death-associated morphological changes are conserved across nematode species and paralyzing treatments. **a.** The death-associated contraction and expansion compared between 113 *C. elegans* and 83 *C. briggsae* nematodes. Both strains were analyzed using a functional data analysis (FDA) to decompose the morphological changes into orthogonal harmonics. The mean trajectory of the first harmonic is plotted for *C. elegans (black solid line)* and *C. briggsae (green solid line)*, along with the +1 and -1 standard deviation as as dashed lines. This representation allows the both population-average trajectory and inter-individual variation between populations to be visalized simultaneously. **b.** The magnitude of death-associated contraction and expansion was quantified for each worm using the PCA loadings from first PCA function. The cumulative distribution function compares the distribution of expansion sizes in *C. elegans (black)* and *C. briggsae (green)* populations. **c-d** The same PCA analysis was performed to compare 113 *C. elegans* to 70 *P. pacificus* nematodes, (e-f) 97 wildtype *C. elegans* to 179 severely paralyzed *C. elegans unc-119 (ed3)* mutants, and (g-h) wildtype *C. elegans* housed either on 0 mM or 9 mM Antimycin A (N=248, N= 166).

1.2 Automated identification of movement and behavioral states using HMM models

We then set out to develop automated means for classifying animals' morphological/behavioral states, and thereby eliminate the laborious by-hand annotation of images. We augmented the lifespan machine software to estimate, in each image collected of each individual, two features describing movement and morphology: an improved "movement score" and a "change in apparent body size" score. The movement score quantifies the magnitude of an individuals' postural change, calculated as the sum of the absolute value of per-pixel changes in consecutive images of that individual. Apparent body size is estimated as the sum of intensities of all pixels corresponding to an individual in a single image. Because the lifespan machine allows nematodes to freely explore their environment, we found that local features on the agar surface adversely affected our ability to accurately estimate the absolute size of an individual. For example, individuals located in shadows cast by the plate appeared smaller, and animals located on top of inhomogeneities in the bacterial lawn appeared larger. Fortunately, we found that the contribution of such environmental features was time-invariant at the timescale of nematode aging, which allowed us to improve our analysis by focusing on changes in individual body size, which differentiated away errors in absolute size estimation. Therefore, we focused on "movement score" and "change in apparent body size" as features on which to classify animals' morphological and behavioral states. To characterize the discriminatory power of our "movement score" and "change in apparent body size" metrics, we compared the distribution of each metric across morphological/behavioral states in our by-hand annotated wild-type data set. We found that each state exhibited a distinct, characteristic set of distributions of movement scores and change in apparent body sizes (Fig B in S1 Text). This suggested that it might be possible to implement a Hidden Markov Model (HMM) to identify morphological/behavioral state transitions from time series of movement scores and change in apparent body size measurements. To obtain probabilistic, predictive estimates of movement state, we fit each state's distributions of "movement scores" and "change in apparent body size" with a two-dimensional Gaussian Mixture Model. To obtain a predictive model of state transition times, we fit the distribution of times spent in each distribution with an exponential model. We then integrated these GMMs using the Viterbi algorithm, which allowed us to models and "solve" an individual's aging trajectory—identifying the most series of state transition times that best explain the observed time series of movement and body size scores.

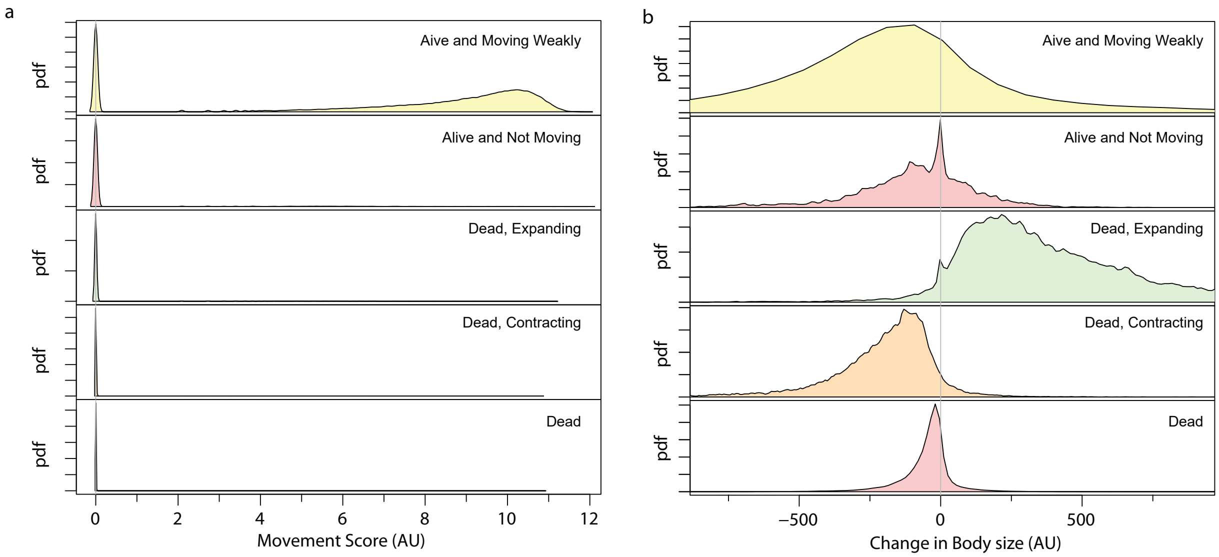

Fig B in S1 Text: Death-associated morphological changes support robust automated identification of death times. a. In an manually-curated data set of 351 individuals, we calculated the empirical probability distribution function for all observations of each animal in each state. **b.** A empirical probability distribution functions quantifying the magnitude of change in apparent body size observed for each animal in each state.

Supporting Text 1 References

1 Stroustrup N, Ulmschneider B, Nash Z, López-Moyado I, Apfeld J, Fontana W. The Caenorhabditis Elegans Lifespan Machine. Nature Methods. 2013;10:665–70.

2 Ragsdale EJ, Müller MR, Rödelsperger C, Sommer RJ. A Developmental Switch Coupled to the Evolution of Plasticity Acts through a Sulfatase. Cell. 2013;155(4):922–933. doi:10.1016/j.cell.2013.09.054.

3 Stein LD, Bao Z, Blasiar D, Blumenthal T, Brent MR, Chen N, et al. The Genome Sequence of Caenorhabditis Briggsae: A Platform for Comparative Genomics. PLoS Biology. 2003;1(2):e45. doi:10.1371/journal.pbio.0000045.

4 Churgin MA, Jung SK, Yu CC, Chen X, Raizen DM, Fang-Yen C. Longitudinal Imaging of Caenorhabditis Elegans in a Microfabricated Device Reveals Variation in Behavioral Decline during Aging. eLife. 2017;6:e26652. doi:10.7554/eLife.26652.
