# Supplementary material for: A hierarchical process model links behavioral aging and lifespan in C. elegans
Source: PLoS Comput Biol. 2022 Sep 30;18(9):e1010415. doi: 10.1371/journal.pcbi.1010415 (PMC9524676; doi:10.1371/journal.pcbi.1010415)
Supplement: S2 Text — A parametric approach to quantifying similarities and differences in the time-dependent dynamics of the risk of vigorous movement cessation and death. (PDF) [file pcbi.1010415.s008.pdf]

Supporting Text 2–Age-associated Risk Trajectories of VMC and death

We find that the risk of VMC and the risk of death increase throughout life. To understand the similarities between these age-associated risk trajectories, we developed a method to quantify the shape of event-specific hazard curves within and across replicates. Exploring a set of four parametric fits previously (Statistical Methods), we found that a three-parameter, frailty-corrected Weibull model, previously shown to accurately model nematode death times[1], provided the best fit for both VMC and death times (Panels a-c in Fig A in S2 Text) across all replicates. Because all replicates shared the same parametric form, we could then compare VMC and death risk trajectories using parametric fits. Across all replicates, we found that vigorous movement curves exhibited a moderately lower Weibull shape (alpha) parameter (Panel e in Fig A in S2 Text), reflecting a moderately slower increase in the risk of ceasing vigorous movement compared to the risk of death. Vigorous movement also exhibited a moderately higher frailty (sigma) parameter (Panel f in Fig A in S2 Text) compared to death, reflecting a more substantial late-life risk deceleration compared to death. We therefore conclude that VMC and death times exhibit similar but not identical aging trajectories. It has been shown that the dynamics of risk trajectories reflect the dynamics of the underlying age-associated physiologic declines that determine risk[2]. Based on this perspective, we can conclude that VMC and death are determined by similar but not identical age-associated physical declines.

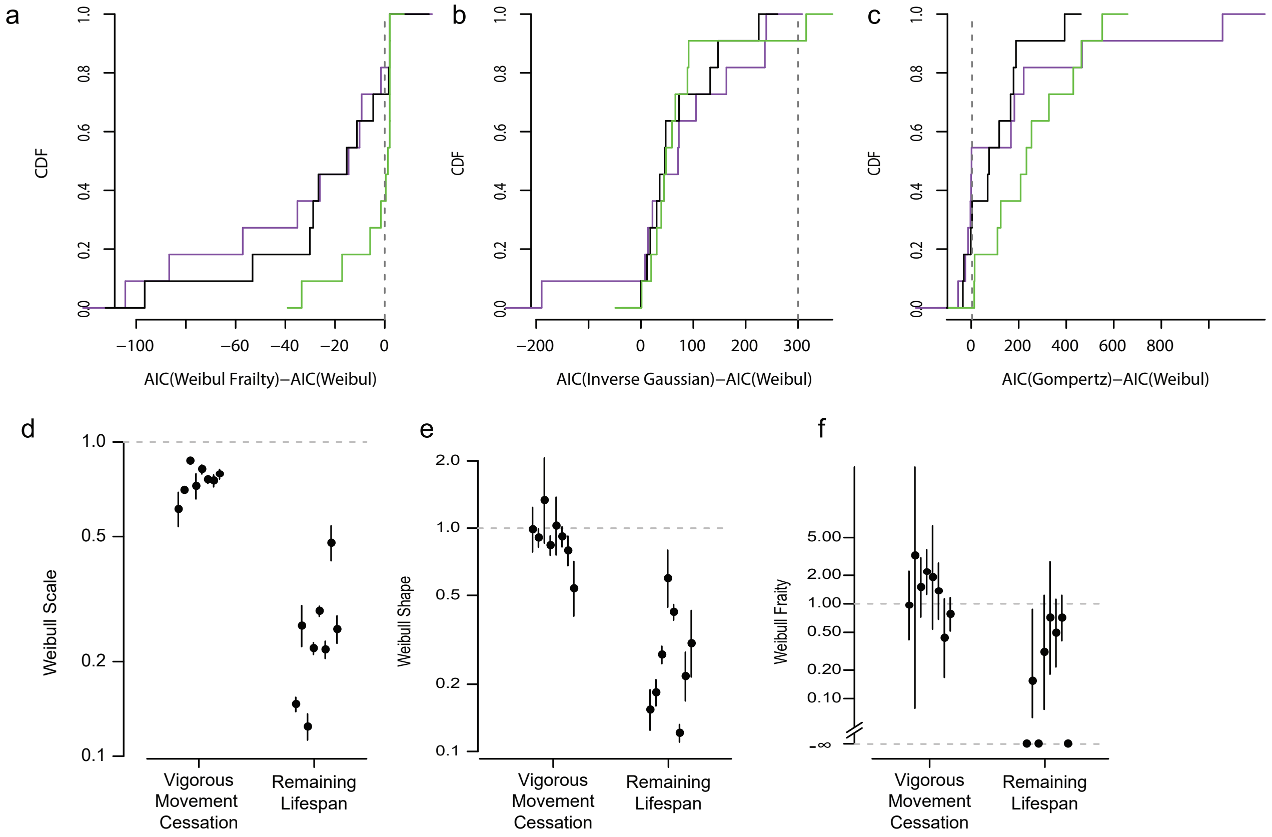

Fig A in S2 Text: Comparing the shape of risk trajectories for VMC and lifespan a. To compare the quality of fit between the three-parameter and two-parameter Weibull models, the cumulative distribution function (CDF) across ten replicates are shown, comparing the AIC for VMC times *(purple)*, for death times *(black)*, and for the duration of life remaining after VMC *(green)*. Negative values indicate a preference for the three parameter Weibull over the two parameter Weibull model. **b.** The same analysis, but comparing the Inverse Gaussian distribution to the two parameter Weibull model. **c.** The same analysis, but comparing the Gompertz distribution to the two-parameter Weibull distribution. **d.** Across all models, VMC times and the durations of life remaining after VMC were fit with three parameter Weibull models. The Weibull scale parameter was compared between the two periods. **e.** The Weibull shape parameter was compared between the two periods. **f.** The Weibull frailty parameter was compared between the two periods.

2 Supporting Text 2 References

1 Stroustrup N, Anthony WE, Nash ZM, Gowda V, Gomez A, López-Moyado IF, et al. The Temporal Scaling of Caenorhabditis Elegans Ageing. Nature. 2016;530(7588):103.

2 Aalen O, Borgan O, Gjessing H. Survival and Event History Analysis : A Process Point of View. New York, NY : Springer New York; 2008.
