# Supplementary material for: A hierarchical process model links behavioral aging and lifespan in C. elegans
Source: PLoS Comput Biol. 2022 Sep 30;18(9):e1010415. doi: 10.1371/journal.pcbi.1010415 (PMC9524676; doi:10.1371/journal.pcbi.1010415)
Supplement: S3 Text — Formal specification of the process model relating vigorous movement cessation and lifespan, and its relationship to the slope of the linear regression line relating vigorous movement cessation and death times. (PDF) [file pcbi.1010415.s009.pdf]

3 Supporting Text 3–Process models of VMC and death times

3.1 A single-state Markov process

We observe two events in each individuals life, v_i and d_i, the timing of vigorous movement cessation and death respectively. We presume that each event arises as the outcome of some time-dependent physical decline. In this section, we seek to understand how our measurement of the distributions V and D inform us about the relationship between the physical declines determining v_i and d_i.

To accomplish this, we introduce a third variable X which we cannot observe directly but infer for each individual i using v_i and d_i. Based on the observed residual structure (S2b Fig) we define X such that $x_i = d_i - v_i$ with $x_i \sim X$. Note that S2b Fig of the main text excludes the alternative model $d_i = \frac{v_i}{x_i}$, as the regression residuals of the proportional model have higher heteroskedasticity than the additive model. Furthermore, note that x_i is never zero and therefore we need not consider that truncation of V by D might influence the relationship between V and D. We diagram this relationship in Fig A in S3 Text.

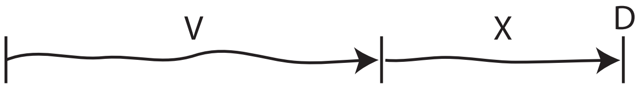

Fig A in S3 Text: A model where V and X run in series

We consider the case where V and X arise as first-passage times across distinct thresholds of a single Markov process. If the Markov process has a single state that determines both v_i and d_i, then all individuals will necessarily share an equivalent state at their VMC times v_i because each will have just passed the same threshold. In consequence, each individual i will have an equivalent remaining lifespan at v_i such that $E(X|V = t)$ becomes independent of t.

Consider the linear regression

$$d_i = \alpha v_i + \epsilon_i + c \tag{1}$$

This regression will estimate a value of α that ensures

$$E(D|V = t) = \alpha t + c \tag{2}$$

with $E(\epsilon|V) = 0$. Because $d_i = v_i + x_i$, it follows by construction that

$$E(D|V = t) = t + E(X|V = t) \tag{3}$$

and therefore by substituting in Eq. 1 we arrive at the expression

$$E(X|V = t) = (\alpha - 1)t + c \tag{4}$$

For a single-state Markov process $E(X|V = t)$ must be independent of t, which will occur only when α equals exactly 1. Because our data shows that, generally, $\alpha < 1$, we conclude that a single-state Markov process model is inconsistent with our data.

3.2 A system of two Markov processes

We therefore consider whether two Markov Process–or a two-dimensional Markov process which is equivalent–is sufficient to explain our data. We find that $\alpha < 1$, which implies that X and V are negatively correlated. To interpret this we must now be more explicit about our model regarding the causal relationship between V and D. Consider two such relationships between V, D, and a potential upstream confounder R.

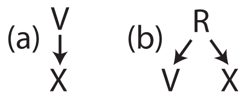

Fig B in S3 Text: Two possible causal relationships between V and X

Causal models (a) and (b) differ in the relationship between V and X: causal in (a) but non-causal in (b). How does $\alpha < 1$ constrain these two models? For model (a), $\alpha < 1$ implies that V must exert some negative influence on X. The biological implications of this are described in the main text

In contrast, model (b) allows the correlation between V and X to arise from the action of some upstream factor R. Because V is no longer causally upstream of X, then the correlation implied by Eq. 1 no longer must be explained by some paradoxical physical interaction between V and X. Instead, the value of $\alpha < 1$ would arise based on the particular form of $E(D|V = t)$ determined incidentally by the two conditional distributions $D|R$ and $V|R$. This can be demonstrated by considering the two extremes: if R fully determines the variability in V and X, then by definition $E(X|V)$ becomes independent of R and therefore independent of time, yielding an $\alpha = 1$, At the opposite extreme, if R has no influence on V and X then V and X become uncorrelated such that $\alpha = 0$. Any realization of model (b) that falls between these extreme cases would then produce an α whose value lies between 0 and 1.

3.3 A system of two Markov processes (in parallel)

The constrains described in the previous section hold not only if the time-dependent processes V and X occur in series, but also they progress in parallel, as shown in Fig C in S3 Text.

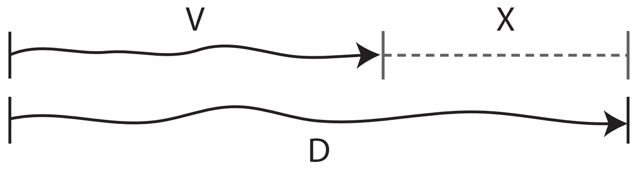

Fig C in S3 Text: A model where V and D run in parallel

Here, $d_i \geq v_i$ remains true only because $E(D) \gg E(V)$ and $E(D) - E(V)$ and the variances of V and D are low enough that the tails of V and D do not overlap. In the case of causal model (a) the process determining V must be coupled to D such that D progresses slower before $t = v_i$ but then faster after $t = d_i$. In contrast, causal model (b) allows V and D to progress in parallel, such that R can have a time-independent influence to act similarly hasten or slow both V and D.

3.4 Summary

We therefore conclude that our regression analysis corresponding to Eq. (1) excludes the possibility that V and D are outcomes of a single-state Markov process, which is the assumption underlying much current work. Instead, a system of two Markov processes–or a single two-dimensional Markov process which is equivalent–allows a single systemic factor to act similarly on the processes determining both D and V to produce the observed correlations and in particular a slope $\alpha < 1$ in Eq. (1).
