# Supplementary material for: A hierarchical process model links behavioral aging and lifespan in C. elegans
Source: PLoS Comput Biol. 2022 Sep 30;18(9):e1010415. doi: 10.1371/journal.pcbi.1010415 (PMC9524676; doi:10.1371/journal.pcbi.1010415)
Supplement: S4 Text — An exploration of alternate hypothesis for why the slope of the linear regression line relating vigorous movement cessation and death times would be less than one. (PDF) [file pcbi.1010415.s010.pdf]

4 Supporting Text 4–Accounting for environmental effects and measurement error

We cannot perfectly observe D or V. Instead, our observations of the "true" D and V are subject to distortion by the confounding effects environmental variations and measurement error. If we represent environmental effects by the factor Z and measurement error by ϵ, we can relate our measured $D*$ and $V*$ to the "true" underlying D and V as follows:

$$\begin{aligned} v_i^* &= v_i + z_{v_i} + \epsilon_{v,i} + c_v \\ d_i^* &= d_i + z_{d_i} + \epsilon_{d,i} + c_d \end{aligned} \tag{1}$$

with $E(\epsilon_{v,i}) = E(\epsilon_{d,i}) = E(z_{v,i}) = E(z_{d,i}) = 0$ and $Var(\epsilon_{v,i}) = \sigma^2_{ev}$, $Var(\epsilon_{d,i}) = \sigma^2_{ed}$, $Var(z_{v,i}) = \sigma^2_{zv}$, and $Var(z_{d,i}) = \sigma^2_{zd}$. In this application, there can be no measurement error in Z, as Z is a categorical variable coding for a discrete organizational unit (the plate or device on which the measurements were obtained) predefined before the start of the study.
In regression Eq. 1 in S3 Text, we seek to estimate the relationship $d_i = \alpha V + \epsilon_{b,i}$. Combining this with 1, we find that the relationship between observed variables $D*$ and $V*$ is

$$d_i^* = \alpha v_i^* - z_{d_i} + \alpha z_{v.i} + \epsilon_{b,i} - \epsilon_{d,i} + \alpha \epsilon_{v,i} - c_d + \alpha * c_v \tag{2}$$

The correlation between v_i, Z, and ϵ_{v_i} is an unfortunate example measurement error's effect on regression. We wish to estimate α in the presence of environmental factors Z using the multiple regression model

$$D_i = \beta_V V_i + \beta_Z X_i + \epsilon_i; \tag{3}$$

However, the influence of α on V, Z and ϵ in Eq. 2 introduces structural problems in such estimation. Multiple regression in the presence of measurement error is well studied (Buonaccorsi. Measurement Error: Models, Methods, and Applications) (E. S. Gillespie. The influence of measurement errors in multiple linear regression.), and predicts that β_V can be a biased estimator of α. The nature of this bias will depend on the correlation between Z and V, as well as the relative magnitudes of σ^2_{ev}, σ^2_{ed}, σ^2_{zv} and σ^2_{zd}. To avoid extended analytic excursions, in the following sections we follow two approaches to identify any bias in our estimation of α using regression 3.

4.1 Bias in multiple regression estimates I : Estimating α within batches

First, we study the relationship between our estimates of α across the whole data set to estimates obtained in subsets of the data. We consider the data presented in Fig 2 of the main text which describes 2346 individuals, organized into four batches. Each batch represents a flatbed scanner, which produces characteristic set of unmeasured environmental factors (e.g temperature) that influence the V and D of all animals housed on that scanner. As previously shown elsewhere, regression 3 estimates α at a value significantly below 1. Without accounting for batch effects, the single regression Eq. 1 in S3 Text fails to identify a significant deviation from $\alpha = 1$, highlighting the importance of accounting for batch effects. To see whether B_Y in the multiple regression 3 is a biased estimator of α, we considered the relationship between D and V in each batch separately, using the single regression model Eq. 1 in S3 Text. In Table A in S4 Text, we find that when grouped with each batch-estimated β_y, the all-batch estimate of β_y is the median value. This suggests that the structure of our multiple regression approach (Eq. 2) does not add substantial bias to our estimate of α on top of what is present in a single variate regression model of the same data.

Data used	Model	N	β_Y	95% CI	p-value (a <1)	Intercept	95% CI
All	$d_i = \beta_V V_i + \beta_Z Z_i$	1380	0.86	(0.82-0.91)	<.0001	4.83	(4.23-5.43)
All	$d_i \beta_V V_i$	1380	0.98	(0.93-1.02)	0.1432	3.49	(2.93-4.05)
Batch 1	$d_i \beta_V V_i$	312	0.82	(0.72-0.92)	0.0006	6.00	(4.73-7.26)
Batch 2	$d_i \beta_V V_i$	430	0.78	(0.70-0.87)	<.0001	6.33	(5.24-7.42)
Batch 3	$d_i \beta_V V_i$	391	0.97	(0.89-1.05)	0.2666	2.49	(1.63-3.35)
Batch 4	$d_i \beta_V V_i$	247	0.89	(0.79-0.98)	0.0054	4.56	(3.36-5.77)

Table A in S4 Text: Comparing α estimates within and across batches

The values of β_D in Table A in S4 Text suggests that our multiple regression approach does not introduce additional bias compared to single regression model estimates. The estimate of β_v obtained via multiple regression is centered among batch-specific β_v estimates.
It still remains the case that these single regression model estimates may themselves be biased due to the measurement error term $\epsilon_{v,i}$ in Eq. 1. Measurement error in single regression produces attenuation in parameter estimates that case might make $|\beta_D| \leq |\alpha|$. This would impede our ability to distinguish between causal models (1), (2), and (3) by preventing us from distinguishing between $\alpha = 1$ or $\alpha < 1$. Fortunately in this case analytic theory is more straightforward than for multiple regression. Attenuation will take the form $|\beta_V| = |\hat{\kappa}\alpha|$ with

$$\hat{\kappa} = \frac{\hat{\sigma}_D^2}{\hat{\sigma}_D^2 + \hat{\sigma}_{Derr}^2} \tag{4}$$

Here, $\hat{\sigma}_V^2$ and $\hat{\sigma}_{Verr}^2$ are the variances of V and the measurement error in V in eq. 1. By substituting in our empiric estimate $\beta_V = 0.8$ and asserting $\alpha = 1$, we can use eq. 4 to predict the relative magnitude of measurement error required to produce a bias large enough to generate the observed deviation from $\beta_v = 1$. We find that $\hat{\sigma}_{Verr}^2 = 0.25\hat{\sigma}_V^2$, so that a measurement error with a standard deviation equal to half of V would be required produce the observed bias. Unfortunately, the nature of our time-to-event data is that repeated measurements are impossible, as each individual's cessation of vigorous movement and death can only be observed once. It's therefore impossible to directly estimate the true empiric ratio of $\hat{\sigma}_{Verr}^2/\hat{\sigma}_V^2$. However, we can inspect various aspects of our data to estimate the contribution of measurement error to our analysis.
In the wild-type data set shown in main text Fig 2, $\hat{\sigma}_V = 2.2$. Numerical simulation using a Weibull distribution V and additive Gaussian noise for measurement error, we found that the underlying biological noise and measurement error would combine to show $\hat{\sigma}_V = 2.2$ when $\sigma_V = 1.99$ and $\sigma_{Verr} = 1.00$, as shown below.

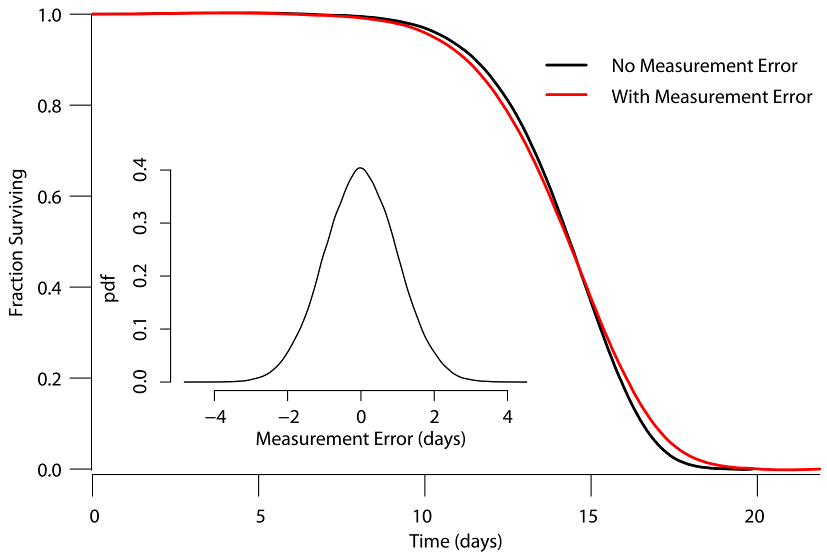

Fig A in S4 Text: Additive error with the standard deviation such that $\hat{\sigma}_{Verr}^2 = 0.25\hat{\sigma}_V^2$ is shown in the PDF inset. This error has the effect of transforming the "true" distribution (black) into an "observed" distribution (red).

Fig A in S4 Text provides an example in which measurements of an individuals' vigorous movement span would need to have an average absolute error of 0.8 days. This is roughly two-fold higher than the typical error of our automated analysis pipeline, and subsequent by-hand annotation of our data sets reduces the error to less than 0.1 days error on average. Yet, reducing error below 0.8 days on average is a taxing standard, and so we performed additional diagnostics to see whether the observed $\hat{\sigma_V}$ or other population level statistics correlated with the observed β_V.

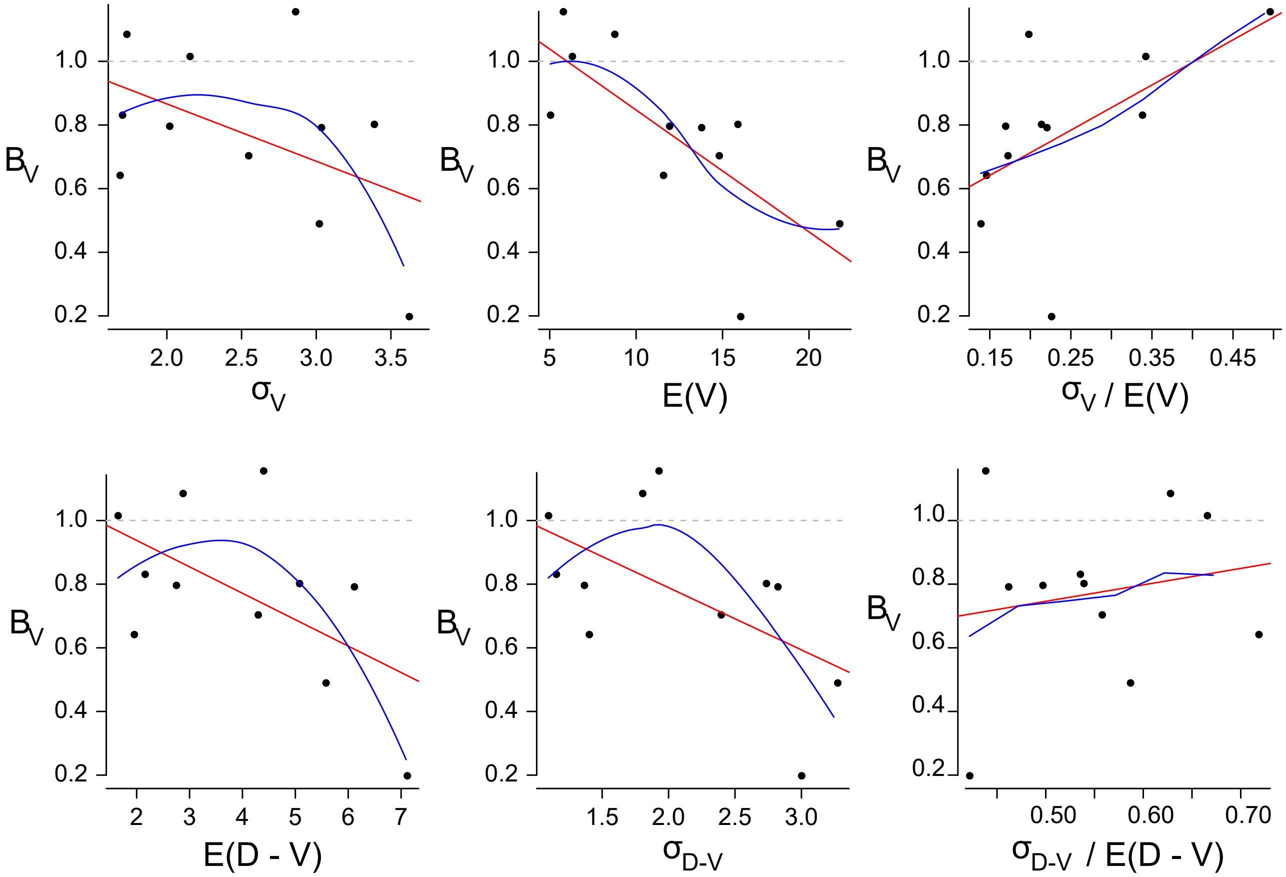

Fig B in S4 Text: The relationship between observed B_v and population summary statistics across replicate experiments

We found that the β_v was inversely correlated with σ_V, but also with the $E(V)$. Were V to vary across replicates by a scaling relationship, we'd expect proportional changes in σ_V and $E(V)$. So, we calculated the coefficient of variation to understand the "mean-normalized" with our β_v estimate. We found that "less noisy" experiments in fact showed lower β_v, not supporting the hypothesis that measurement-error is attenuating our parameter estimates. We then considered the relationship between D and V and β_V. Larger intervals v_i d_i correlated with smaller B_v, but so did the standard error $\hat{\sigma}^2_{D-V} = var(d_i - v_i)$. So, we calculated the coefficient of variation for $D - V$, and found no relationship with B_v estimates. In conclusion, though we identified relationships between β_V and various population statistics including σ_V, accounting for the understood mean-variance relationship between distributions of vigorous movement span, increased variation is related to increased (not attenuated) β_v estimates.
Therefore, though we cannot fully exclude the role of measurement error, and eagerly await improvements in measurement accuracy that will definitively resolve the issue, we find no evidence to substantiate theoretic concerns that measurement errors make β_v deviate from α in our analysis.

4.2 Simulating the effect of measurement error and environmental effects

To demonstrate our ability to distinguish between causal models (2) and (3) in the presence of environmental noise, we performed a series of simulations.

Variables shared in both simulations

$$\begin{aligned} V^\dagger &\sim Weibull(\beta = 12, \alpha = 5.75) \\ R &\sim Gamma(\beta = 70, \alpha = 1/70) \\ Z &\in \{1, 2, 3, \ldots, N\} \\ \beta_Z &\sim Gamma(\beta = 50, \alpha = 1/50) \end{aligned} \tag{5}$$

Simulation of Causal Model (2)

$$\begin{aligned} X^\dagger &\sim Weibull(\beta = 3, \alpha = 2.5) \\ v_i &= r_i * v_i^\dagger \\ x_i &= x_i * x_i^\dagger \\ d_i &= v_i + x_i \\ v_i^* &= v_i * (\beta_Z Z_i) \\ d_i^* &= d_i * (\beta_Z Z_i) \end{aligned} \tag{6}$$

Simulation of Causal Model (3)

$$\begin{aligned} D^\dagger &\sim Weibull(\beta = 15, \alpha = 7.2) \\ v_i &= r_i v^\dagger \\ d_i &= r_i d^\dagger \\ v_i^* &= v_i * \beta_Z Z_i \\ d_i^* &= d_i * \beta_Z Z_i \end{aligned} \tag{7}$$

The "true" underlying sources of biological variability $V^\dagger$, $X^\dagger$, and $D^\dagger$ are modeled as Weibull distributions with parameters chosen to approximate our empiric data. The causal effect of R is modeled by multiplying each individual's "true" v_i and x_i (model (2)) or d_i (model (3)) by a value drawn from a gamma distribution with a mean of 1. R thereby introduces a correlation between V X, and D in model (2), and between V and D in model (3). Batch effects, meant to mimic the influence of environmental factors, were introduced by randomly assigning each individual i membership to a batch Z. Each batch produces a characteristic effect shared among individuals in that batch. The "observed" V^* and D^* analyzed in the simulation are obtained by multiplying v_i and d_i by the batch effect $\beta_Z Z_i$ characteristic of batch Z_i. Note that here we choose to model noise and environmental variation as multiplicative factors in contrast to previous sections, as multiplicative noise is likely to more closely match the physical effect of temperature and other factors on event times (Stroustrup et al 2016.)

First, we performed a simulation in which paired v_i and d_i were generated as samples from the V and D as defined above for each model. The regression model Eq. 1 in S3 Text was fit to estimate β_v, allowing us to evaluate the performance of our statistical approach in the absence of batch effects. Then, we performed the same simulation but this time comparing v_i^* and d_j^* generated as samples from V^* and ${}^D*$ defined above for each model. The regression model 3 was fit to estimate β_v, allowing us to evaluate the performance of our statistical approach in the presence of environmental confounders. In both sets of simulations, 1000 replicates were performed each considering a population of 1000 simulated individuals. In addition to estimates of β_v, we obtained bootstrapped p-values for the test $H_0 : \beta_v < 1$. We summarize these results in Table B in S4 Text, providing the average β_v obtained across all replicates as well as the fraction of p-values obtained that fell below a $p = .05$ threshold.

Data Generating Model	Batch effects added?	β_v	Fraction of replicates with significant $\alpha < 1$ at $p < .05$
Model (2)	No	0.999	.044
Model (2)	Yes	1.000	.056
Model (3)	No	.520	1.000
Model (3)	Yes	.519	0.985

Table B in S4 Text: Differentiating simulated causal models.

These results show that regardless of environmental effects, we can differentiate between causal models (1) and (2). Causal model (2) generates data matching the expected relationship of $D = \alpha V + \epsilon$ with $\alpha = 1$. Our false positive rate of our bootstrapping test for $\beta_v! = 1$ matches the expected rate of 5%. Causal model (3) generates data matching the expected relationship $D = \alpha V + \epsilon$ with $\alpha < 1$. The with a false negative rate at this effect magnitude appears at or below 2.5%.

4.3 Power Simulation

To estimate our power to identify $\alpha \neq 1$ and the sample sizes required to detect the deviations we observe, we performed a simulation. Using the data presented in Fig 2 of the main text which describes 2346 wildtype individuals, we sub-sampled the data set, selecting an equal number of individuals from each batch (device). Across 1000 replicates at each sample size, we then measured the relationship between β_v when estimated using a single regression model Eq. 1 in S3 Text or the full multiple regression model 2. In each replicate, we calculated the boot-strapped p-value for $\beta_v! = 1$ for the single and multiple regression models, and estimated the proportion of replicates for which this p-value was significant at $p < .05$

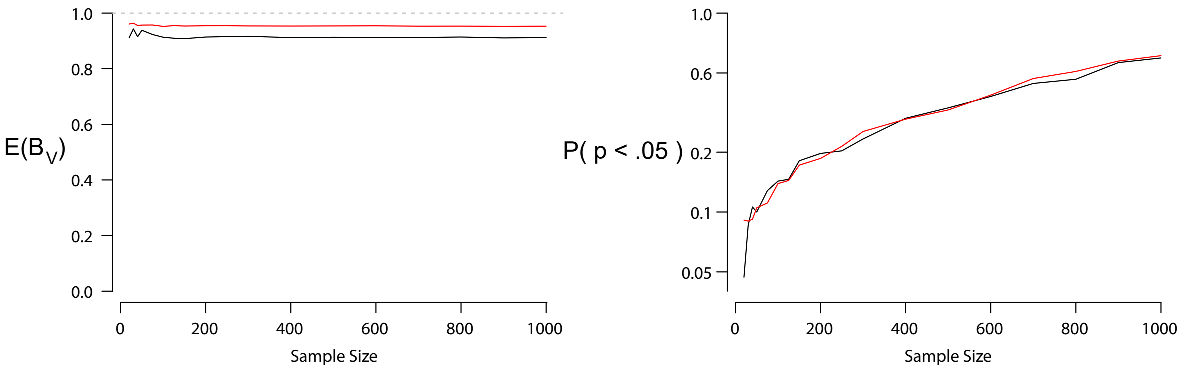

Fig C in S4 Text: Subsampling our empiric data to estimate parameter bias and statistical power as a function of sample size. Multiple regression to account for batch effects (black) and the naive single regression (red).

We found that failure to account for batch effects biased the estimation of β_v toward 1 at all population sizes. However, given the small overall magnitude of effect, large populations were required to obtain marginal statistical power, with a power of 0.60 being reached only when 1000 individuals were considered.
