# Supplementary material for: A hierarchical process model links behavioral aging and lifespan in C. elegans
Source: PLoS Comput Biol. 2022 Sep 30;18(9):e1010415. doi: 10.1371/journal.pcbi.1010415 (PMC9524676; doi:10.1371/journal.pcbi.1010415)
Supplement: S5 Text — A description of the regression model used to characterize the differential effects of interventions on vigorous movement cessation and lifespan. (PDF) [file pcbi.1010415.s011.pdf]

5 Supporting Text 5–Quantifying proportional and disproportional effects of interventions on lifespan and VMC

5.1 A formal definition for proportionate and disproportionate effects of interventions on VMC and lifespan

In our hierarchical process model, interventions can alter three entities: the aging process that determines VMC, the aging process that determines death times, and the shared upstream factor that influences the rate of both processes. In this section, we develop a model to estimate, from data, the relative effect a mutation or intervention has on each process individually compared to the shared effect it has on both via the upstream factor.

The effects we model serve to re-scale vigorous movement, V and death D distributions. This does not depend on any particular form of the joint distribution of V and D, and so we can simplify the task of statistical inference by treating V and D as independent. We therefore model V and D as endpoints of two Wiener processes with a shared drift rate parameter μ_s

$$\begin{aligned} Pv_t &= (\mu_v \mu_s)t + \sigma W_t \\ Pd_t &= (\mu_d \mu_s)t + \sigma W_t \end{aligned} \tag{1}$$

In this case, μ_s represents the influence of the upstream shared factor in our hierarchical process model. Note that both processes share the same σ, another simplifying assumption that we take because it is emperically justified–we observe scaling (or very close to it) between V and D across the different interventions and mutations

The first passage times of Weiner processes like Pv_t and Pd_t are known to take the form inverse-gaussian-distributions:

$$\begin{aligned} P_v &\sim invgauss(mean = \frac{R}{\mu_v \mu_s}, shape = (\frac{R}{\sigma})^2) \\ P_d &\sim invgauss(mean = \frac{R}{(\mu_d \mu_s)}, shape = (\frac{R}{\sigma})^2) \end{aligned} \tag{2}$$

We can describe the effect of a mutation or intervention on these end-point distributions via its effect on the process-specific drift terms, $\Delta\mu_v$ and $\Delta\mu_d$, and the shared drift term$\Delta\mu_s$:

$$\begin{aligned} P_{v,\text{wildtype}} &\sim invgauss(mean = \frac{R}{\mu_v \mu_s}, shape = (\frac{R}{\sigma})^2) \\ P_{d,\text{wildtype}} &\sim invgauss(mean = \frac{R}{\mu_d \mu_s}, shape = (\frac{R}{\sigma})^2) \\ P_{v,\text{intervention}} &\sim invgauss(mean = \frac{R}{\mu_v \mu_s \Delta\mu_v \Delta\mu_s}, shape = (\frac{R}{\sigma})^2) \\ P_{d,\text{intervention}} &\sim invgauss(mean = \frac{R}{\mu_d \mu_s \Delta\mu_d \Delta\mu_s}, shape = (\frac{R}{\sigma})^2) \end{aligned} \tag{3}$$

With Eq. 3, we are close to being able to formally describe the proportionate and disproportionate effects of interventions on VMC and lifespan in terms of parameters that can be estimated from data. The proportional effects are summarized by the value of $Delta\mu_s$ of each intervention, and the disproportionate effects are summarized by $\Delta\mu_v$ and $\Delta\mu_d$.

Since we are not concerned with the absolute magnitude of the drift terms in our process model–the absolute rates of aging–but rather the relative rates of aging compared between genotypes or before and after interventions, we fix R to 1.
We now have a model whose parameters can be estimated using semi-parametric Accelerated Failure Time regression:

$$\begin{aligned} log(v_{\text{intervention}_i}) &= \beta_v v_{\text{wildtype},i} + \epsilon_{v,i} \\ log(d_{\text{intervention}_i}) &= \beta_d d_{\text{wildtype},i} + \epsilon_{d,i} \end{aligned} \tag{4}$$

In Eq. 4, the β parameters serve to event time data to the corresponding data-generating Weiner processes according to:

$$\begin{aligned} \beta_v &= log(\Delta\mu_v \Delta\mu_s) \\ \beta_d &= log(\Delta\mu_d \Delta\mu_s) \end{aligned} \tag{5}$$

From this definition of β_v and β_d, we see that $\frac{\Delta\mu_v}{\Delta\mu_d} = \frac{\beta_d}{\beta_v}$, irrespective of the value of $\Delta\mu_s$.
Therefore $\Delta\mu_s$ functions to set the shared scale of both $\Delta\mu_v$ and $\Delta\mu_v$ without influencing their ratio.
This ratio quantifies the disproportionate effect of interventions on vigorous movement and lifespan, so it is important enough to give it its own symbol:

$$R_f = \frac{\Delta\mu_v}{\Delta\mu_d} \tag{6}$$

5.2 Estimating proportionate and disproportionate effects from VMC and lifespan data

This formal definition has the advantage of using quantities that can be directly estimated from our data, using an AFT regression model:

$$log(y_i) = \beta_t t_i + \beta_x x_i + \beta_C t_i x_i + \epsilon_i \tag{7}$$

where y_i is the event time drawn the set $Y = \{V, D\}$, i.e the set including both VMC and death times with t_i as a categorical variable specifying whether y_i is a VMC or death time. X specifies the population in which y_i was observed, either wild-type or intervention-exposed population. In this model, the cross term β_C provides an estimate of the ratio R_f (the disproportionate action of an intervention on V and D) and β_x provides $\Delta\mu_s$ (the proportionate action of an intervention on V and D).

In S5 Fig, we compare the estimates of R_f and $\Delta\mu_s$ for each intervention and mutation considered in Fig 5 of the main text. Note that Eq. 7 provides us with the formal language needed to state the coarse graining model described in the main text as an empirically-testable hypothesis: coarse-graining is appropriate when interventions act mostly proportionally on vigorous movement and lifespan, such that $|\beta_x| \gg 1$ while $R_f \approx 1$ In other words, coarse-graining is appropriate when a data set is well fit by non-zero values of $\Delta\mu_s$, with fine effects summarized by relatively small deviations from $R_f = 1$.

In S5 Fig we present the parameter estimates of this model fit across the set of mutants and interventions, and observe that many interventions show large β_x estimates and R_f estimates very close to one. In Fig 6 d of the main text, we present the parameter estimates of this model fit to the α-Naphthaleneacetic acid dosage series, and see similar changes for R_f in both *rpb-2::AID* and *rpb-2(+)* strains, but large changes in β_x only for the *rpb-2::AID* strain.
